# Variational Bayesian identification and prediction of stochastic nonlinear dynamic causal models

**DOI:** 10.1016/j.physd.2009.08.002

**Published:** 2009-11-01

**Authors:** J. Daunizeau, K.J. Friston, S.J. Kiebel

**Affiliations:** Wellcome Trust Centre for Neuroimaging, University College, London, United Kingdom

**Keywords:** Approximate inference, Model comparison, Variational Bayes, EM, Laplace approximation, Free-energy, SDE, Nonlinear stochastic dynamical systems, Nonlinear state-space models, DCM, Kalman filter, Rauch smoother

## Abstract

In this paper, we describe a general variational Bayesian approach for approximate inference on nonlinear stochastic dynamic models. This scheme extends established approximate inference on hidden-states to cover: (i) nonlinear evolution and observation functions, (ii) unknown parameters and (precision) hyperparameters and (iii) model comparison and prediction under uncertainty. Model identification or inversion entails the estimation of the marginal likelihood or evidence of a model. This difficult integration problem can be finessed by optimising a free-energy bound on the evidence using results from variational calculus. This yields a deterministic update scheme that optimises an approximation to the posterior density on the unknown model variables. We derive such a variational Bayesian scheme in the context of nonlinear stochastic dynamic hierarchical models, for both model identification and time-series prediction. The computational complexity of the scheme is comparable to that of an extended Kalman filter, which is critical when inverting high dimensional models or long time-series. Using Monte-Carlo simulations, we assess the estimation efficiency of this variational Bayesian approach using three stochastic variants of chaotic dynamic systems. We also demonstrate the model comparison capabilities of the method, its self-consistency and its predictive power.

## 1 Introduction

In nature, the most interesting dynamical systems are only observable through a complex (and generally non-invertible) mapping from the system’s states to some measurements. For example, we cannot observe the time-varying electrophysiological states of the brain but we can measure the electrical field it generates on the scalp using electroencephalography (EEG). Given a model of neural dynamics, it is possible to estimate parameters of interest (such as initial conditions or synaptic connection strengths) using probabilistic methods (see e.g. [1], or [2]). However, incomplete or imperfect model specification can result in misleading parameter estimates, particularly if random or stochastic forces on system’s states are ignored [3]. Many dynamical systems are nonlinear and stochastic; for example neuronal activity is driven by, at least partly, physiological noise (see e.g. [4,5]). This makes recovery of both neuronal dynamics and the parameters of their associated models a challenging focus of ongoing research (see e.g. [6,7]). Another example of stochastic nonlinear system identification is weather forecasting; where model inversion allows predictions of hidden-states from meteorological models (e.g. [8]). This class of problems is found in many applied research fields such as control engineering, speech recognition, meteorology, oceanography, ecology and quantitative finance. In brief, the identification and prediction of stochastic nonlinear dynamical systems have to cope with subtle forms of uncertainty arising from; (i) the complexity of the dynamical behaviour of the system, (ii) our lack of knowledge about its structure and (iii) our inability to directly measure its states (hence the name “hidden- states”). This speaks to the importance of probabilistic methods for identifying nonlinear stochastic dynamic models (see [9] for a “data assimilation” perspective).

Most statistical inference methods for stochastic dynamical systems rely on a state-space formulation i.e. the specification of two densities; the likelihood, derived from an observation model and a first-order Markovian transition density, which embodies prior beliefs about the evolution of the system [10]. The nonlinear filtering and smoothing^1^ problems have already been solved using a Bayesian formulation by Kushner [11] and Pardoux [12] respectively. These authors show that the posterior densities on hidden-states given the data so far (filtering) or all the data (smoothing) obey stochastic partial differential (Kushner–Pardoux) equations. However:

• They suffer from the curse of dimensionality; i.e. an exponential growth of computational complexity with the number of hidden-states [13]. This is why most approximate inversion techniques are variants of the simpler Kalman filter [14,15] or [10,16]. Sampling based approximations to the posterior density (particle filters, see e.g. [58] or [17]) have also been developed, but these also suffer from the curse of dimensionality.
• The likelihood and the transition densities depend on the potentially unknown parameters and hyperparameters^2^ of the underlying state-space model. These quantities have also to be estimated and induce a hierarchical inversion problem, for which there is no generally accepted solution (see [18] for an approximate maximum-likelihood approach to this problem). This is due to the complexity (e.g. multimodality and high-order dependencies) of the joint posterior density over hidden-states, parameters and hyperparameters. The hierarchical structure of the generative model prevents us from using the Kushner–Pardoux equations or Kalman Filter based approximations. A review of modified Kalman filters for joint estimation of model parameters and hidden-states can be found in Wan [19].
These issues make variational Bayesian (VB) schemes [20–23] appealing candidates for joint estimation of states, parameters and hyperparameters. However, somewhat surprisingly, only a few VB methods have been proposed to finesse this triple estimation problem for nonlinear systems. These include:
• Roweis and Ghahramani [24] propose an Expectation-Maximization algorithm that yields an approximate posterior density over hidden-states and maximum-likelihood estimates of the parameters.
• Valpola and Karhunen [25] propose a VB method for unsupervised extraction of dynamic processes from noisy data. The nonlinear mappings in the model are represented using multilayer perceptron networks. This dynamical blind deconvolution approach generalizes [24], by deriving an approximate posterior density over the mapping parameters. However, as in Roweis [24] the method cannot embed prior knowledge about the functional form of both observation and evolution processes.
• Friston et al. [7], present a VB inversion scheme for nonlinear stochastic dynamical models in generalized coordinates of motion. The approach rests on formulating the free-energy optimization dynamically (in generalized coordinates) and furnishes a continuous analogue to extended Kalman smoothing algorithms. Unlike previous schemes, the algorithm can deal with serially correlated state-noise and can optimize a joint posterior density on all unknown quantities.

Despite the advances in model inversion described in theses papers, there remain some key outstanding issues: First, the difficult problem of time-series prediction, given the (inferred) structure of the system (see [26] for an elegant Gaussian process solution). Second, no attempt has been made to assess the statistical efficiency of the proposed VB estimators for nonlinear systems (see [27] for a study of asymptotic behaviour of VB estimators for conjugate-exponential models). Third, there has been no attempt to optimize the form or structure of the state-space model using approximate Bayesian model comparison.

In this paper, we present a VB approach for approximating the posterior density over hidden-states and model parameters of stochastic nonlinear dynamic models. This is important because it allows one to infer the hidden-states causing data, parameters causing the dynamics of hidden-states and any non-controlled exogenous input to the system, given observations. Critically, we can make inferences even when both the observation and evolution function are nonlinear. Alternatively, this approach can be viewed as an extension of VB inversion of static models (e.g. [28]) to invert nonlinear state-space models. We also extend the VB scheme to approximate both the predictive density (on hidden-states and measurement space) and the sojourn density (i.e. the stationary distribution of the Markov chain) that summaries long-term behaviour [29].

In brief, model inversion entails optimizing an approximate posterior density that is parameterized by its sufficient statistics. This density is derived by updating the sufficient statistics using an iterative coordinate ascent on a free-energy bound on the marginal likelihood. We demonstrate the performances of this VB inference scheme when inverting (and predicting) stochastic variants of chaotic dynamic systems.

This paper comprises three sections. In the first, we review the general problem of model inversion and comparison in a variational Bayesian framework. More precisely, this section describes the extension of the VB approach to non-Gaussian posterior densities, under the Laplace approximation. The second section demonstrates the VB-Laplace update rules for a specific yet broad class of generative models, namely: stochastic dynamic causal models (see [1] for a Bayesian treatment of deterministic DCMs). It also provides a computationally efficient alternative to the standard tool for long-term prediction (the stationary or sojourn density), based upon an approximation to the predictive density. The third section provides an evaluation of the method’s capabilities in terms of accuracy, model comparison, self-consistency and prediction, using Monte Carlo simulations from three stochastic nonlinear dynamical systems. In particular, we compare the VB approach to standard extended Kalman filtering, which is used routinely in nonlinear filtering applications. We also include results providing evidence for the asymptotic efficiency of the VB estimator in this context. Finally, we discuss the properties of the VB approach.

## 2 Approximate variational Bayesian inference

### 2.1 Variational learning

To interpret any observed data y with a view to making predictions based upon it, we need to select the best model m that provides formal constraints on the way those data were generated; and will be generated in the future. This selection can be based on Bayesian probability theory to choose among several models in the light of data. This necessarily involves evaluating the marginal likelihood; i.e. the plausibility of observed data given model m:

(1) p(y|m)=∫p(y,ϑ|m)dϑ

where the generative model m is defined in terms of a likelihood p(y|ϑ,m) and prior p(ϑ|m) on the model parameters, ϑ, whose product yields the joint density by Bayes rule:

(2) p(y,ϑ|m)=p(y|ϑ,m)p(ϑ|m).

The marginal likelihood or evidence p(y|m) is required to compare different models. Usually, the evidence is estimated by converting the difficult integration problem in Eq. (1) into an easier optimization problem by optimizing a free-energy bound on the log-evidence. This bound is constructed using Jensen’s inequality and is induced by an arbitrary density q(ϑ)[21]:

(3) F(q,y)=lnp(y|m)−D=U−S

$$D=\int q\left(\vartheta \right)\ln\frac{q\left(\vartheta \right)}{p\left(\vartheta |y,m\right)}d\vartheta \text{.}$$

The free-energy comprises an energy term $U={\langle \ln p\left(y,\vartheta \right)\rangle }_{q}$ and an entropy term $S={\langle \ln q\left(\vartheta \right)\rangle }_{q}$.^3^ The free-energy is a lower bound on the log-evidence because the Kullback–Leibler cross-entropy or divergence, D between the arbitrary and posterior densities is non-negative. Maximizing the free-energy with respect to q(ϑ) minimizes the divergence, rendering the arbitrary density q(ϑ)≈p(ϑ|y,m) an approximate posterior density.

To make this maximization easier one usually assumes q(ϑ) factorizes into approximate marginal posterior densities, over sets of parameters $\vartheta_{i}$

(4) $$q\left(\vartheta \right)=\underset{i}{\prod }q_{i}\left(\vartheta_{i}\right)\text{.}$$

In statistical physics this is called a mean-field approximation [30]. This approximation replaces stochastic dependencies between the partitioned model variables by deterministic relationships between the sufficient statistics of their approximate marginal posterior density (see [31] and below).

Under the mean-field approximation it is straightforward to show that the approximate marginal posterior densities satisfy the following set of equations [32]:

(5) $$\frac{\delta F}{\delta q}=0\Rightarrow q\left(\vartheta_{i}|\lambda_{i}\right)=\frac{1}{Z_{i}}\exp\left(I\left(\vartheta_{i}\right)\right)$$

$$I\left(\vartheta_{i}\right)=\int \underset{j\neq 1}{\prod }d\vartheta_{j}q_{j}\left(\vartheta_{j}|\lambda_{j}\right)\ln p\left(\vartheta ,y|m\right)$$

where $\lambda_{i}$ are the sufficient statistics of the approximate marginal posterior density $q_{i}$, and $Z_{i}$ is a normalisation constant (i.e., partition function). We will call $I\left(\vartheta_{i}\right)$ the variational energy. If the integral in Eq. (5) is analytically tractable (e.g., through the use of conjugate priors) the above Boltzmann equation can be used as an update rule for the sufficient statistics. Iterating these updates then provides a simple deterministic optimization of the free-energy with respect to the approximate posterior density.

### 2.2 The Laplace approximation

When inverting realistic generative models, nonlinearities in the likelihood function generally induce posterior densities that are not in the conjugate-exponential family. This means that there are an infinite number of sufficient statistics of the approximate posterior density; rendering the integral in Eq. (5) analytically intractable. The Laplace approximation is a useful and generic device, which can finesse this problem by reducing the set of sufficient statistics of the approximate posterior density to its first two moments. This means that each approximate marginal posterior density is further approximated by a Gaussian density:

(6) $$q\left(\vartheta_{i}|\lambda_{i}\right)\approx N\left(\lambda_{i}\right):\lambda_{i}=\left(\begin{array}{c} \mu_{i}=\langle \vartheta_{i}\rangle \\ \Sigma_{i}=\langle \left(\vartheta_{i}-\mu_{i}\right){\left(\vartheta_{i}-\mu_{i}\right)}^{T}\rangle \end{array}\right)$$

where the sufficient statistics $\lambda_{i}=\left(\mu_{i},\Sigma_{i}\right)$ encode the posterior mean and covariance of the i-th approximate marginal posterior density. This (fixed-form) Gaussian approximation is derived from a second-order truncation of the Taylor series to the variational energy [28]:

(7) $$\mu_{i}=\underset{\vartheta_{i}}{\arg\max}I\left(\vartheta_{i}\right)$$

$$\Sigma_{i}=-{\left[{\frac{\partial^{2}}{\partial \vartheta_{i}^{2}}I\left(\vartheta_{i}\right)|}_{\vartheta_{i}=\mu_{i}}\right]}^{-1}$$

$$I\left(\vartheta_{i}\right)\approx L\left(\vartheta_{i},\mu_{\setminus i}\right)+\underset{j\neq i}{\sum }\mathrm{tr}\left[{\frac{\partial^{2}}{\partial \overset{2}{\underset{j}{\vartheta }}}L\left(\vartheta_{i},\vartheta_{j}\right)|}_{\vartheta_{j}=\mu_{j}}\Sigma_{j}\right]$$

L(ϑ)=lnp(ϑ,y|m).

Eq. (7) defines each variational energy and approximate marginal posterior density as explicit functions of the sufficient statistics of the other approximate marginal posterior densities. Under the VB-Laplace approximation, the iterative update of the sufficient statistics just requires the gradients and curvatures of L(ϑ) (the log-joint density) with respect to the unknown variables of the generative model. We will refer to this approximate Bayesian inference scheme to as the VB-Laplace approach.

### 2.3 Statistical Bayesian inference

The VB-Laplace approach above provides an approximation q(ϑ) to the posterior density p(ϑ|y,m) over any unknown model parameter ϑ, given a set of observations y and a generative model m. Since this density summarizes our knowledge (from both the data and priors), we could use it as the basis for posterior inference; however, these densities generally tell us more than we need to know. In this section, we briefly discuss standard approaches for summarizing such distributions; i.e. Bayesian analogues for common frequentist techniques of point estimation and confidence interval estimation.^4^ We refer the reader to [33] for further discussion.

To obtain a point estimate $\overset{ˆ}{\vartheta }$ of any unknown we need to select a summary of q(ϑ), such as its mean or mode. These estimators can be motivated by different estimation losses, which, under the Laplace approximation, are all equivalent and reduce to the first-order posterior moment or posterior mean. The Bayesian analogue of a frequentist confidence interval is defined formally as follows: a 100×(1−π)% posterior confidence interval for ϑ is a subset C of the parameter space, such that its posterior probability is equal to 1−π; i.e., $1-\pi =\int_{C}q\left(\vartheta \right)d\vartheta$. Under the Laplace approximation, the optimal 100×(1−π)% posterior confidence interval is the interval whose bounds are the π/2 and 1−π/2 quantiles of q(ϑ)[34]. This means Bayesian confidence intervals are simple functions of the second-order posterior moment or posterior variance. We will demonstrate this later.

In what follows, we introduce the class of generative models we are interested in; i.e. hierarchical stochastic nonlinear dynamic models. We then present update equations for each approximate marginal posterior density, starting with the straightforward updates (the parameters of the generative model) and finishing with the computationally more demanding updates of the time-varying hidden-states. These are derived from a variational extended Kalman–Rauch marginalization procedure [10], which exploits the Laplace approximation above.

## 3 Variational Bayesian treatment of stochastic DCMs

In this section, we illustrate VB inference in the context of an important and broad class of generative models. These are stochastic dynamic causal models that combine nonlinear stochastic differential equations governing the evolution of hidden-states and a nonlinear observer function, to provide a nonlinear state-space model of data. Critically, neither the states nor the parameters of the state-space model functions are known. This means that the generative model is hierarchical, which induces a natural mean-field partition into states and parameters. This section describes stochastic DCMs and the update rules entailed by our VB-Laplace approach. In the next section, we illustrate the performance of the method in terms of model inversion, selection and time-series prediction using Monte Carlo simulations of chaotic systems.

### 3.1 Stochastic DCMs and state-space models

The generative model of a stochastic DCM rests on two equations: the observation equation, which links observed data $y_{1:T}$ comprising T vector-samples to hidden-states $x_{t}$ and a stochastic differential equation (SDE) governing the evolution of these hidden-states:

(8) $$y_{t}=g\left(x_{t},\varphi ,u_{t},t\right)+\varepsilon_{t}$$

$$dx_{t}=a\left(x_{t},\theta ,u_{t},t\right)dt+b\left(x_{t},t\right)d\varpi_{t}$$

where φ and θ are unknown parameters of the observation function g and equation of motion (drift) a respectively; $u_{t}$ are known exogenous inputs that drive the hidden-states or response; $\varepsilon_{t}\in {ℜ}^{p\times 1}$ is a vector of random Gaussian measurement-noise; b may, in general, be a function of the states and time and $\varpi_{t}$ denotes a Wiener process or state-noise that acts as a stochastic forcing term.

A Wiener process is a continuous zero mean random process, whose variance grows as time increases; i.e. 

(9) $$\langle \varpi_{t}\rangle =0\text{,}\;\langle {\left(\varpi_{s}-\varpi_{t}\right)}^{2}\rangle =s-t:\;0\leq s\leq t\text{.}$$

The continuous-time formulation of the SDE in Eq. (8) can also be written using the following (stochastic) integral formulation:

(10) $$x_{t+\Delta t}=x_{t}+\underset{\text{Riemann integral}}{\underset{︸}{\int_{t}^{t+\Delta t}a\left(x_{t},\theta ,u_{t},t\right)dt}}+\underset{\text{Ito’s integral}}{\underset{︸}{\int_{t}^{t+\Delta t}b\left(x_{t},t\right)d\varpi_{t}}}$$

where the second integral is a stochastic integral, whose peculiar properties led to the derivation of Ito stochastic calculus [35]. Eq. (10) can be converted into a discrete-time analogue using local linearization, or Euler–Maruyama methods, yielding the standard first-order autoregressive process (AR(1)) form of nonlinear state-space models:

(11) $$y_{t}=g\left(x_{t},\varphi ,u_{t},t\right)+\varepsilon_{t}$$

$$x_{t+1}=f\left(x_{t},\theta ,u_{t},t\right)+\eta_{t}$$

where $\eta_{t}\in {ℜ}^{n\times 1}$ is a Gaussian state-noise vector of variance $b^{2}\Delta t$ and f is the evolution function given by:

(12) $$f\left(x_{t},\theta ,u_{t},t\right)\approx x_{t}+J{\left(x_{t}\right)}^{-1}\left(\exp\left[J\left(x_{t}\right)\Delta t\right]-I_{n}\right)a\left(x_{t},\theta ,u_{t},t\right)\overset{}{\underset{\Delta t\to 0}{⟶}}x_{t}+\Delta t\;a\left(x_{t},\theta ,u_{t},t\right)\text{.}$$

Here J is the Jacobian of a and Δt is the time interval between samples. The first line corresponds to the local linearization method [36], and the second line instantiates the so-called Euler–Maruyama discretisation scheme [35]. The discrete-time variant of the state-space model yields the Gaussian likelihood and transition densities (where dependence on exogenous inputs and time is left implicit):

(13) $$p\left(y_{t}|x_{t},\varphi ,\sigma ,m\right)=N\left(g\left(x_{t},\varphi \right),\sigma^{-1}I_{p}\right)$$

$$p\left(x_{t+1}|x_{t},\theta ,\alpha ,m\right)=N\left(f\left(x_{t},\theta \right),\alpha^{-1}I_{n}\right)$$

where σ (resp. α) is the precision of the measurement-noise $\varepsilon_{t}$ (resp. state-noise $\eta_{t}$). From Eqs. (10) and (13), we note that the state-noise precision is $\alpha ={\left(b^{2}\Delta t\right)}^{-1}$, where the transition density can be regarded as a prior that prescribes the likely evolution of hidden-states. From now on, we will assume the state-noise precision is independent of the hidden-states, which narrows the class of generative models we deal with (e.g. GARCH models, see [37]); volatility models, see e.g. [38]; bilinear stochastic models, see [39].

#### 3.1.1 The predictive and sojourn densities

The predictive density over the hidden-states is derived from the transition density given in Eq. (13) through the iterated Chapman–Kolmogorov equation:

(14) $$p\left(x_{t}|x_{0},\theta ,\alpha ,m\right)=\int \cdots \int \overset{t}{\underset{k=1}{\prod }}p\left(x_{k}|x_{k-1},\theta ,\alpha ,m\right)dx_{k-1}\propto \int \cdots \int \exp\left[-\frac{\alpha }{2}\overset{t}{\underset{k=1}{\sum }}{\left(x_{k}-f\left(x_{k-1},\theta \right)\right)}^{2}\right]\overset{t}{\underset{k=1}{\prod }}dx_{k-1}\text{.}$$

This exploits the Markov property of the hidden-states. Despite the Gaussian form of the transition density, nonlinearities in the evolution function render the predictive density non-Gaussian. In particular, nonlinear evolution functions can lead to multimodal predictive densities.

Under mild conditions, it is known that nonlinear stochastic systems as in Eq. (8) are ergodic, i.e. their distribution becomes stationary [40]. The fact that a dynamical system is ergodic means that random state-noise completely change its stability properties. Its deterministic variant can have several stable fixed points or attractors, whereas, when there are stochastic forces, there is a unique steady state, which is approached in time by all other states. Any local instabilities of the deterministic system disappear, manifesting themselves only in the detailed form of the stationary density. This (equilibrium) stationary density, which we will call the *sojourn density*, is given by the predictive density when t→∞. The sojourn density summarizes the long-term behaviour of the hidden-states: it quantifies the proportion of time spent by the system at each point in state-space (the so-called “sojourn time”). We will provide approximate solutions to the sojourn density below and use it in the next section for long-term prediction.

#### 3.1.2 The hierarchical generative model

In a Bayesian setting, we also have to specify prior densities on the unknown parameters of the generative model m. Without loss of generality,^5^ we assume Gaussian priors on the parameters, initial conditions of the hidden-states and Gamma priors on the precision hyperparameters:

(15) $$p\left(x_{0}|m\right)=N\left(\varsigma_{0},\upsilon_{0}\right)$$

$$p\left(\varphi |m\right)=N\left(\varsigma_{\varphi },\upsilon_{\varphi }\right)$$

$$p\left(\theta |m\right)=N\left(\varsigma_{\theta },\upsilon_{\theta }\right)$$

$$p\left(\sigma |m\right)=Ga\left(\varsigma_{\sigma },\upsilon_{\sigma }\right)$$

$$p\left(\alpha |m\right)=Ga\left(\varsigma_{\alpha },\upsilon_{\alpha }\right)\text{,}$$

where $\varsigma_{\varphi },\upsilon_{\varphi }$ (*resp.* $\varsigma_{\theta },\upsilon_{\theta }$ and $\varsigma_{0},\upsilon_{0}$) are the prior mean and covariance of the observation parameters φ (*resp.* the evolution parameters θ and initial condition $x_{0}$); and $\varsigma_{\sigma },\upsilon_{\sigma }$ (*resp*. $\varsigma_{\alpha },\upsilon_{\alpha }$) are the prior shape and inverse scale parameters of the Gamma-variate precision of the measurement-noise (*resp.* state-noise).

**Fig. 1:**
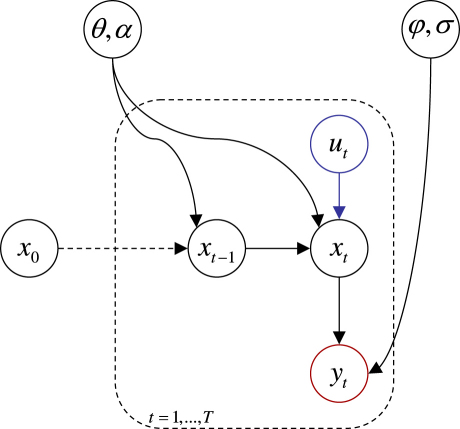
Graph representing the generative model m: The sequence of observations $y_{1:T}$ is represented as the plate over T pairs of hidden variables $x_{1:T}$ ($x_{0}$ denotes the initial condition of the hidden-states). φ and θ are unknown parameters of the observation and evolution function. $u_{1:T}$ is an exogenous input. σ (resp. α) is the precision (inverse variance) of the unknown measurement-noise $\varepsilon_{t}$ (resp. unknown state-noise $\eta_{t}$).

Fig. 1 shows the Bayesian dependency graph representing the ensuing generative model defined by Eqs. (13) and (15). The structure of the generative model is identical to that in [22]; the only difference is the nonlinearity in the observation and evolution functions (i.e. in the likelihood and transition densities). This class of generative model defines a stochastic DCM and generalizes both static convolution models (i.e. $f\left(x_{t},\theta \right)=0$) and non-stochastic DCMs (i.e. α→∞).

### 3.2 The VB-Laplace update rules

The mean-field approximation to the approximate posterior density, for the state-space model m described above is

(16) $$q\left(\vartheta \right)=\underset{i}{\prod }q\left(\vartheta_{i}\right)=q\left(\varphi \right)q\left(\theta \right)q\left(\sigma \right)q\left(\alpha \right)q\left(x_{1:T}\right)q\left(x_{0}\right)\text{.}$$

Eq. (5) provides the variational energy of each mean-field partition variable using the expectations of L(ϑ)=logp(ϑ,y|m), under the Markov blanket^6^ of each of these variables. Using the mean-field partition in Eq. (16), these respective variational energies are (omitting constants for clarity):

(17) $$I\left(\varphi \right)={\langle L\left(\varphi ,\sigma ,x\right)\rangle }_{q\left(\sigma \right)q\left(x_{1:T}\right)}$$

$$I\left(\theta \right)={\langle L\left(\theta ,\alpha ,x\right)\rangle }_{q\left(\alpha \right)q\left(x_{1:T}\right)q\left(x_{0}\right)}$$

$$I\left(\sigma \right)={\langle L\left(\sigma ,\varphi ,x\right)\rangle }_{q\left(\varphi \right)q\left(x_{1:T}\right)}$$

$$I\left(\alpha \right)={\langle L\left(\alpha ,\theta ,x\right)\rangle }_{q\left(\theta \right)q\left(x_{1:T}\right)q\left(x_{0}\right)}$$

$$I\left(x_{1:T}\right)={\langle L\left(x_{0:T},\sigma ,\varphi ,\alpha ,\theta \right)\rangle }_{q\left(\sigma \right)q\left(\varphi \right)q\left(\alpha \right)q\left(\theta \right)q\left(x_{0}\right)}$$

$$I\left(x_{0}\right)={\langle L\left(x_{0:1},\alpha ,\theta \right)\rangle }_{q\left(\alpha \right)q\left(\theta \right)q\left(x_{1:T}\right)}\text{.}$$

We will use the VB-Laplace approximation (Eq. (7)) to handle nonlinearities in the generative model when deriving approximate posterior densities, with the exception of the precision hyperparameters, for which we used free-form VB update rules.

#### 3.2.1 Updating the sufficient statistics of the hyperparameters

Under the VB-Laplace approximation on the parameters and hidden-states, the approximate posterior density of the precision parameters (α,σ) does not require any further approximation. This is because their prior is conjugate to a Gaussian likelihood. Therefore, their associated VB update rule is derived from the standard free-form approximate posterior density in Eq. (5).

First, consider the free-form approximate posterior density of the measurement-noise precision. It can be shown that q(σ) has the form $\ln q\left(\sigma \right)=\left(a_{\sigma }-1\right)\ln\left(\sigma \right)-b_{\sigma }\sigma +c$, which means q(σ) is a Gamma density

(18) $$q\left(\sigma \right)=Ga\left(a_{\sigma },b_{\sigma }\right)\Rightarrow \mu_{\sigma }=\frac{a_{\sigma }}{b_{\sigma }}$$

with shape and scale parameters $a_{\sigma },b_{\sigma }$ given by

(19) $$a_{\sigma }=\frac{1}{2}\left(2\varsigma_{\sigma }+pT\right)$$

$$b_{\sigma }=\frac{1}{2}\left(2\upsilon_{\sigma }+\mathrm{tr}\left[{\overset{ˆ}{\varepsilon }}_{1:T}^{T}\;{\overset{ˆ}{\varepsilon }}_{1:T}\right]+\overset{T}{\underset{t=1}{\sum }}\mathrm{tr}\left[\left(\frac{\partial g}{\partial \varphi }{\frac{\partial g}{\partial \varphi }}^{T}+\frac{\partial^{2}\overset{˜}{g}}{\partial x\partial \varphi }\left(I_{p}⊗\Psi_{t,t}\right)\frac{\partial^{2}{\overset{˜}{g}}^{T}}{\partial x\partial \varphi }\right)\Sigma_{\varphi }\right]+\overset{T}{\underset{t=1}{\sum }}\mathrm{tr}\left[\frac{\partial g}{\partial x}{\frac{\partial g}{\partial x}}^{T}\Psi_{t,t}\right]\right)\text{.}$$

Here, ${\overset{ˆ}{\varepsilon }}_{1:T}$ is a p×T matrix of prediction errors in measurement space; ${\overset{ˆ}{\varepsilon }}_{t}=g\left(\mu_{x,t},\mu_{\varphi }\right)-y_{t}$, and $\Psi_{t,t}$ denotes the n×n instantaneous posterior covariance of the hidden-states (see below). A similar treatment shows that α is also *a posteriori* Gamma-distributed:

(20) $$q\left(\alpha \right)=Ga\left(a_{\alpha },b_{\alpha }\right)\Rightarrow \mu_{\alpha }=\frac{a_{\alpha }}{b_{\alpha }}$$

with shape and scale parameters

(21) $$a_{\alpha }=\frac{1}{2}\left(2\varsigma_{\alpha }+nT\right)$$

$$b_{\alpha }=\frac{1}{2}\left(2\upsilon_{\alpha }+\mathrm{tr}\left[{\overset{ˆ}{\eta }}_{1:T}^{T}\;{\overset{ˆ}{\eta }}_{1:T}\right]+\overset{T-1}{\underset{t=1}{\sum }}\mathrm{tr}\left[\left(\frac{\partial f}{\partial \theta }{\frac{\partial f}{\partial \theta }}^{T}+\frac{\partial^{2}\overset{˜}{f}}{\partial x\partial \theta }\left(I_{n}⊗\Psi_{t,t}\right)\frac{\partial^{2}{\overset{˜}{f}}^{T}}{\partial x\partial \theta }\right)\Sigma_{\theta }\right]+\overset{T-1}{\underset{t=1}{\sum }}\mathrm{tr}\left[\left(I_{n}+\frac{\partial f}{\partial x}{\frac{\partial f}{\partial x}}^{T}\right)\Psi_{t,t}\right]+\mathrm{tr}\left[\frac{\partial f}{\partial x}{\frac{\partial f}{\partial x}}^{T}\Psi_{0,0}+\Psi_{T,T}\right]-2\overset{T-1}{\underset{t=1}{\sum }}\mathrm{tr}\left[{\frac{\partial f}{\partial x}}^{T}\Psi_{t,t+1}\right]\right)$$

where ${\overset{ˆ}{\eta }}_{t}=f\left(\mu_{x,t},\mu_{\theta }\right)-\mu_{x,t-1}$ is the n×1 vector of estimated state-noise, $\Psi_{t,t+1}$ is the n×n lagged posterior covariance of the hidden-states (see below).

#### 3.2.2 Updating the sufficient statistics of the parameters

These updates follow the same procedure above, except that the VB-Laplace update rules for deriving the approximate posterior densities of the parameters are based on an iterative Gauss–Newton optimization of their respective variational energy (see Eqs. (6) and (7)). Consider the variational energy of the observation parameters:

(22) $$I\left(\varphi \right)={\langle L\left(\varphi ,\sigma ,x\right)\rangle }_{q\left(\sigma \right)q\left(x\right)}\approx \left(L\left(\varphi ,\mu_{x},\mu_{\sigma }\right)+\frac{1}{2}\mathrm{tr}\left[\frac{\partial^{2}}{\partial x^{2}}L\left(\varphi ,\mu_{x},\mu_{\sigma }\right)\Sigma_{x}\right]\right)\approx \left(\frac{\partial L}{\partial \varphi }+\frac{1}{2}\frac{\partial }{\partial \varphi }\mathrm{tr}\left[\frac{\partial^{2}L}{\partial x^{2}}\Sigma_{x}\right]\right)\left(\varphi -\mu_{\varphi }\right)+{\left(\varphi -\mu_{\varphi }\right)}^{T}\left(\frac{\partial^{2}L}{\partial \varphi^{2}}+\frac{1}{2}\frac{\partial^{2}}{\partial \varphi^{2}}\mathrm{tr}\left[\frac{\partial^{2}L}{\partial x^{2}}\Sigma_{x}\right]\right)\left(\varphi -\mu_{\varphi }\right)\text{.}$$

This quadratic form in φ yield the Gauss–Newton update rule for the mean of the approximate posterior density over observation parameters:

(23) $$\Delta \mu_{\varphi }=\Sigma_{\varphi }\left(\frac{\partial L}{\partial \varphi }+\frac{1}{2}\frac{\partial }{\partial \varphi }\mathrm{tr}\left[\frac{\partial^{2}L}{\partial x^{2}}\Sigma_{x}\right]\right)$$

$$\Sigma_{\varphi }={\left(\frac{\partial^{2}L}{\partial \varphi^{2}}+\frac{1}{2}\frac{\partial^{2}}{\partial \varphi^{2}}\mathrm{tr}\left[\frac{\partial^{2}L}{\partial x^{2}}\Sigma_{x}\right]\right)}^{-1}$$

where the gradient and curvatures are evaluated at the previous estimate of the approximate posterior mean $\mu_{\varphi }$. Note that, in the following, we use condensed notations for mixed derivatives; i.e. 

(24) $$\frac{\partial^{2}\overset{˜}{g}}{\partial \varphi \;\partial x}=\frac{\partial }{\partial \varphi }\mathrm{vec}\left(\frac{\partial g}{\partial x}\right)\text{,}\;\frac{\partial^{2}\overset{˜}{f}}{\partial \theta \;\partial x}=\frac{\partial }{\partial \theta }\mathrm{vec}\left(\frac{\partial f}{\partial x}\right)\text{.}$$

Using a bilinear Taylor expansion of the observation function, Eq. (23) can be implemented as:

(25) $$\Delta \mu_{\varphi }=\Sigma_{\varphi }\left(\upsilon_{\varphi }^{-1}\left(\varsigma_{\varphi }-\mu_{\varphi }\right)+\overset{ˆ}{\sigma }\overset{T}{\underset{t=1}{\sum }}\left(\frac{\partial g}{\partial \varphi }{\overset{ˆ}{\varepsilon }}_{t}-\frac{\partial^{2}\overset{˜}{g}}{\partial \varphi \;\partial x}\left(I_{p}⊗\Psi_{t,t}\right)\;\frac{\partial \overset{˜}{g}}{\partial x}\right)\right)$$

$$\Sigma_{\varphi }={\left(\overset{ˆ}{\sigma }\overset{T}{\underset{t=1}{\sum }}\left(\frac{\partial g}{\partial \varphi }{\frac{\partial g}{\partial \varphi }}^{T}+\frac{\partial^{2}\overset{˜}{g}}{\partial \varphi \;\partial x}\left(I_{p}⊗\Psi_{t,t}\right){\frac{\partial^{2}\overset{˜}{g}}{\partial \varphi \;\partial x}}^{T}\right)+\upsilon_{\varphi }^{-1}\right)}^{-1}\text{.}$$

Similar considerations give the VB-Laplace update rules for the evolution parameters:

(26) $$\Delta \mu_{\theta }=+\Sigma_{\theta }\left(\frac{\partial L}{\partial \theta }+\frac{1}{2}\frac{\partial }{\partial \theta }\mathrm{tr}\left[\frac{\partial^{2}L}{\partial x^{2}}\Sigma_{x}\right]\right)$$

$$\Sigma_{\theta }={\left(\frac{\partial^{2}L}{\partial \theta^{2}}+\frac{1}{2}\frac{\partial^{2}}{\partial \theta^{2}}\mathrm{tr}\left[\frac{\partial^{2}L}{\partial x^{2}}\Sigma_{x}\right]\right)}^{-1}$$

which yields:

(27) $$\Delta \mu_{\theta }=\Sigma_{\theta }\left(\upsilon_{\theta }^{-1}\left(\varsigma_{\theta }-\mu_{\theta }\right)+\overset{ˆ}{\alpha }\overset{T-1}{\underset{t=0}{\sum }}\left(\frac{\partial f}{\partial \theta }{\overset{ˆ}{\eta }}_{t+1}+\frac{\partial^{2}\overset{˜}{f}}{\partial \theta \;\partial x}\left(\mathrm{vec}\left(\Psi_{t,t+1}\right)-\left(I_{n}⊗\Psi_{t,t}\right)\;\frac{\partial \overset{˜}{f}}{\partial x}\right)\right)\right)$$

$$\Sigma_{\theta }={\left(\overset{ˆ}{\alpha }\overset{T}{\underset{t=1}{\sum }}\left(\frac{\partial f}{\partial \theta }{\frac{\partial f}{\partial \theta }}^{T}+\frac{\partial^{2}\overset{˜}{f}}{\partial \theta \;\partial x}\left(I_{n}⊗\Psi_{t,t}\right){\frac{\partial^{2}\overset{˜}{f}}{\partial \theta \;\partial x}}^{T}\right)+\upsilon_{\theta }^{-1}\right)}^{-1}\text{.}$$

Iterating Eqs. (25) and (27) implements a standard Gauss–Newton scheme for optimizing the variational energy of the observation and evolution parameters. To ensure convergence, we halve the size of the Gauss–Newton update until the variational energy increases. Under certain mild assumptions, this regularized Gauss–Newton scheme is guaranteed to converge [41].

#### 3.2.3 Updating the sufficient statistics of the hidden-states

The last approximate posterior density is $q\left(x_{0:T}\right)$. This approximate posterior could be obtained by treating the time-series of hidden-states $x_{0:T}$ as a single finite-dimensional vector and using the VB-Laplace approximation with an expansion of the evolution and observation functions around the last mean. However, it is computationally more expedient to exploit the Markov properties of the dynamics and assemble the sufficient statistics $\mu_{x}$ and $\Sigma_{x}$ sequentially, using a VB-Laplace variant of the extended Kalman–Rauch smoother [10]. These probabilistic filters evaluate the (instantaneous) marginals, $p\left(x_{t}|y_{1:T}\right)$ time point by time point, as opposed to the full joint posterior density over the whole time sequence, $p\left(x_{1:T}|y_{1:T}\right)$. They are approximate solutions to the Kushner–Pardoux partial differential equations that describe the instantaneous evolution of the marginal posterior density on the hidden-states.

Algorithmically, the VB-Laplace Kalman–Rauch marginalization procedure is divided into two passes that propagate (in time) the first and second-order moments of the approximate posterior density. These propagation equations require only the gradients and mixed derivatives of the evolution and observation functions. The two passes comprise a forward pass (which furnishes the approximate filtering density, which can be used to derive an on-line version of the algorithm) and a backward pass (which derives the approximated posterior density from the approximate filtering density).

##### 3.2.3.1 Forward pass

The forward pass entails two steps (prediction and update) that are alternated from t=1 to t=T: The *prediction step* is derived from the Chapman–Kolmogorov belief propagation Eq. (14):

(28) $$\alpha_{t}^{*}\left(x_{t}\right)\propto \int \alpha_{t-1}\left(x_{t-1}\right)\exp\langle \ln p\left(x_{t}|x_{t-1},\theta ,\alpha \right)\rangle dx_{t-1}\overset{q\left(\theta \right)\to \delta \left(\theta \right)}{⟶}p\left(x_{t}|y_{1:t-1}\right)$$

where $\alpha_{t}^{*}\left(x_{t}\right)$ is the current approximate predictive density and $\alpha_{t-1}\left(x_{t-1}\right)$ is the last VB-Laplace approximate filtering density (see above update step). Under the VB-Laplace approximation, the prediction step is given by the following Gauss–Newton update for the predicted mean and covariance:

(29) $$m_{t}^{*}=\underset{\mathrm{standard Gauss}\text{–}\mathrm{Newton EKF prediction}}{\underset{︸}{f\left(\mu_{t-1}^{\left(k\right)},\mu_{\theta }\right)+{\frac{\partial f}{\partial x}}^{T}\left(m_{t-1}-\mu_{t-1}^{\left(k\right)}\right)}}+\underset{\mathrm{mean}\text{-}\mathrm{field perturbation term}}{\underset{︸}{{\overset{ˆ}{\alpha }}^{2}R_{t|t-1}{\frac{\partial f}{\partial x}}^{T}\overset{-1}{\underset{t-1}{B}}\frac{\partial^{2}\overset{˜}{f}}{\partial x\partial \theta }\left(I_{n}⊗\Sigma_{\theta }\right)\;\left({\frac{\partial^{2}\overset{˜}{f}}{\partial x\partial \theta }}^{T}\left(\mu_{t-1}^{\left(k\right)}-m_{t-1}\right)-\frac{\partial \overset{˜}{f}}{\partial \theta }\right)}}$$

$$R_{t|t-1}={\overset{ˆ}{\alpha }}^{-1}{\left(I-\overset{ˆ}{\alpha }{\frac{\partial f}{\partial x}}^{T}B_{t-1}^{-1}\frac{\partial f}{\partial x}\right)}^{-1}$$

$$B_{t-1}=R_{t-1|t-1}^{-1}+\overset{ˆ}{\alpha }\left(\frac{\partial f}{\partial x}{\frac{\partial f}{\partial x}}^{T}+\underset{\mathrm{mean}\text{-}\mathrm{field perturbation term}}{\underset{︸}{\frac{\partial^{2}\overset{˜}{f}}{\partial x\partial \theta }\left(I_{n}⊗\Sigma_{\theta }\right){\frac{\partial^{2}\overset{˜}{f}}{\partial x\partial \theta }}^{T}}}\right)\text{.}$$

This VB-Laplace approximation to the predictive density differs from the traditional extended Kalman filter because it accounts for the uncertainty in the evolution parameters θ (mean-field terms in Eq. (29)). This is critical when making predictions of highly nonlinear systems (as we will see in the next section) with unknown parameters. The *update step* can be written as follows:

(30) $$\alpha_{t}\left(x_{t}\right)\propto \alpha_{t}^{*}\left(x_{t}\right)\;\exp\langle \ln p\left(y_{t}|x_{t},\varphi ,\sigma \right)\rangle \overset{q\left(\varphi \right)\to \delta \left(\varphi \right)}{⟶}p\left(x_{t}|y_{1:t}\right)\text{.}$$

Again, under the VB-Laplace approximation, the update rule for the sufficient statistics of the approximate filtering density is given by:

(31) $$m_{t}=\underset{\mathrm{standard Gauss}\text{-}\mathrm{Newton EKF update}}{\underset{︸}{m_{t}^{*}+\overset{ˆ}{\sigma }R_{t|t}\frac{\partial g}{\partial x}\left(y_{t}-g\left(\mu_{t},\mu_{\varphi }\right)+{\frac{\partial g}{\partial x}}^{T}\left(\mu_{t}-m_{t}^{*}\right)\right)}}+\underset{\mathrm{mean}\text{-}\mathrm{field perturbation term}}{\underset{︸}{\overset{ˆ}{\sigma }R_{t|t}\frac{\partial^{2}\overset{˜}{g}}{\partial x\partial \varphi }\left(I_{p}⊗\Sigma_{\varphi }\right)\;\left({\frac{\partial^{2}\overset{˜}{g}}{\partial x\partial \varphi }}^{T}\left(\overset{\left(k\right)}{\underset{t}{\mu }}-m_{t}^{*}\right)-\frac{\partial \overset{˜}{g}}{\partial \varphi }\right)}}$$

$$R_{t|t}={\left(R_{t|t-1}^{-1}+\overset{ˆ}{\sigma }\left(\frac{\partial g}{\partial x}{\frac{\partial g}{\partial x}}^{T}+\underset{\mathrm{mean}\text{-}\mathrm{field perturbation term}}{\underset{︸}{\frac{\partial^{2}\overset{˜}{g}}{\partial x\partial \varphi }\left(I_{p}⊗\Sigma_{\varphi }\right){\frac{\partial^{2}\overset{˜}{g}}{\partial x\partial \varphi }}^{T}}}\right)\right)}^{-1}\text{.}$$

##### 3.2.3.2 Backward pass

In its parallel implementation (two-filter Kalman–Rauch–Striebel smoother), the backward pass also requires two steps, which are alternated from t=T to t=1. The first is a β-*message passing* scheme:

(32) βt−1(xt−1)∝∫βt(xt)exp〈lnp(xt|xt−1,θ,α)+lnp(yt|xt,φ,σ)〉dxt⟶q(θ)→δ(θ)q(φ)→δ(φ)p(yt+1:T|xt)

Where a local VB-Laplace approximation ensures (omitting constants):

(33) $$\ln\beta_{t}\left(x_{t}\right)=-\frac{1}{2}{\left(x_{t}-n_{t}\right)}^{T}\Omega_{t}^{-1}\left(x_{t}-n_{t}\right)$$

leading to the following mean and covariance backward propagation equation:

(34) $$n_{t-1}=\mu_{t-1}+\overset{ˆ}{\alpha }\;\Omega_{t-1}\left(\frac{\partial f}{\partial x}\left(\mu_{t}-f\left(\mu_{t-1},\mu_{\theta }\right)\right)+\frac{\partial^{2}\overset{˜}{f}}{\partial x\partial \theta }\left(I_{n}⊗\Sigma_{\theta }\right)\frac{\partial \overset{˜}{f}}{\partial \theta }+\frac{\partial f}{\partial x}E_{t}^{-1}\left(\Omega_{t}^{-1}\left(n_{t}-\mu_{t}\right)+\overset{ˆ}{\alpha }\left(f\left(\mu_{t-1},\mu_{\theta }\right)-\mu_{t}\right)-\overset{ˆ}{\sigma }\left(\;\;\frac{\partial g}{\partial x}\left(g\left(\mu_{t},\mu_{\varphi }\right)-y_{t}\right)-\frac{\partial^{2}\overset{˜}{g}}{\partial x\partial \varphi }\left(I_{p}⊗\Sigma_{\varphi }\right)\frac{\partial \overset{˜}{g}}{\partial \varphi }\right)\right)\right)$$

$$\Omega_{t-1}={\overset{ˆ}{\alpha }}^{-1}{\left(\frac{\partial f}{\partial x}{\frac{\partial f}{\partial x}}^{T}+\frac{\partial^{2}\overset{˜}{f}}{\partial x\partial \theta }\left(I_{n}⊗\Sigma_{\theta }\right){\frac{\partial^{2}\overset{˜}{f}}{\partial x\partial \theta }}^{T}-\overset{ˆ}{\alpha }\frac{\partial f}{\partial x}E_{t}^{-1}{\frac{\partial f}{\partial x}}^{T}\right)}^{-1}$$

$$E_{t}=\Omega_{t}^{-1}+\overset{ˆ}{\alpha }I_{n}+\overset{ˆ}{\sigma }\left(\frac{\partial g}{\partial x}{\frac{\partial g}{\partial x}}^{T}+\frac{\partial^{2}\overset{˜}{g}}{\partial x\partial \varphi }\left(I_{p}⊗\Sigma_{\varphi }\right){\frac{\partial^{2}\overset{˜}{g}}{\partial x\partial \varphi }}^{T}\right)\text{.}$$

Note that the β-message is not a density over the hidden-states; it has the form of a likelihood function. More precisely, it is the approximate likelihood of the current hidden-states with respect to all future observations. It contains the information discarded by the forward pass, relative to the approximate posterior density. The latter is given by combining the output of the forward pass (updated density) with the β-message (see below) giving the αβ-*message passing* scheme:

(35) q(xt|y1:T)∝αt(xt)βt(xt)≈N(μt,Ψt,t)⟶q(θ)→δ(θ)q(φ)→δ(φ)p(xt|y1:T)

with, by convention $\beta_{T}\left(x_{T}\right)=1$ and:

(36) $$\mu_{t}=\Psi_{t,t}\left(R_{t}^{-1}m_{t}+\Omega_{t}^{-1}n_{t}\right)$$

$$\Psi_{t,t}={\left(R_{t}^{-1}+\Omega_{t}^{-1}\right)}^{-1}$$

where the necessary sufficient statistics are given in Eqs. (29), (31) and (34). These specify the instantaneous posterior density on the hidden-states.

Eqs. (29), (31), (34) and (36) specify the VB-Laplace update rules for the sufficient statistics of the approximate posterior of the hidden-states. These correspond to a Gauss–Newton scheme for optimizing their variational energy, where the Gauss–Newton increment $\Delta \mu_{1:T}$ is simply the difference between the result of Eq. (36) and the previous approximate mean.

Finally, we need the expression for the lagged posterior covariance $\Psi_{t,t+1}$ to update the evolution, observation and precision parameters (see Eqs. (22) and (25)). This is derived from the following joint density [22]:

(37) $$p\left(x_{t},x_{t+1}|y_{1:T}\right)\propto p\left(x_{t}|y_{1:t}\right)p\left(x_{t+1}|x_{t}\right)p\left(y_{t+1}|x_{t+1}\right)p\left(y_{t+2:T}|x_{t+1}\right)=\alpha_{t}\left(x_{t}\right)\;p\left(x_{t+1}|x_{t}\right)p\left(y_{t+1}|x_{t+1}\right)\beta_{t+1}\left(x_{t+1}\right)\overset{VB}{⟶}\alpha_{t}\left(x_{t}\right)\;\exp\langle p\left(x_{t+1}|x_{t}\right)p\left(y_{t+1}|x_{t+1}\right)\rangle \beta_{t+1}\left(x_{t+1}\right)\approx N\left(\left(\begin{array}{c} \mu_{t}\\ \mu_{t+1} \end{array}\right),\left[\begin{array}{cc} \Psi_{t,t} & \Psi_{t,t+1}\\ \overset{T}{\underset{t,t+1}{\Psi }} & \Psi_{t+1,t+1} \end{array}\right]\right)$$

where the last line follows from the VB-Laplace approximation. As in the forward step of the VB-Laplace Kalman filter, the sufficient statistics of this approximate joint posterior density can be derived explicitly from the gradients of the evolution function:

(38) $$\Psi_{t,t+1}=B_{t}^{-1}\frac{\partial f}{\partial x}{\left({\overset{ˆ}{\alpha }}^{-1}E_{t+1}-\overset{ˆ}{\alpha }{\frac{\partial f}{\partial x}}^{T}B_{t}^{-1}\frac{\partial f}{\partial x}\right)}^{-1}$$

where $E_{t}$ and $B_{t}$ are given in Eqs. (26) and (31), and the gradients are evaluated at the mode $\mu_{1:T}$.

##### 3.2.3.3 Initial conditions

The approximate posterior density over the initial conditions is obtained from the usual VB-Laplace approach. The update rule for the Gauss–Newton optimization of the variational energy of the initial conditions is^7^:

(39) $$\Delta \mu_{0}=\Sigma_{0}\left(\upsilon_{0}^{-1}\left(\varsigma_{0}-\mu_{0}\right)+\overset{ˆ}{\alpha }\left(\frac{\partial f}{\partial x}\left(\mu_{1}-f\left(\mu_{0},\mu_{\theta }\right)\right)-\frac{\partial^{2}\overset{˜}{f}}{\partial x\partial \theta }\left(I_{n}⊗\Sigma_{\theta }\right)\frac{\partial \overset{˜}{f}}{\partial \theta }\right)\right)$$

$$\Sigma_{0}={\left(\overset{ˆ}{\alpha }\left(\frac{\partial f}{\partial x}{\frac{\partial f}{\partial x}}^{T}+\frac{\partial^{2}\overset{˜}{f}}{\partial x\partial \theta }\left(I_{n}⊗\Sigma_{\theta }\right){\frac{\partial^{2}\overset{˜}{f}}{\partial x\partial \theta }}^{T}\right)+\upsilon_{0}^{-1}\right)}^{-1}\text{.}$$

#### 3.2.4 Evaluation of the free-energy

Under the mean-field approximation, the free-energy evaluation requires the sum of the entropy of each approximate marginal posterior density. Except for the hidden-states, evaluating these are relatively straightforward under the Laplace assumption. However, due to the use of the Kalman–Rauch marginalization scheme in the derivation of the posterior $q\left(x_{t}\right)$, the calculation of the joint entropy over the hidden-states requires special consideration. First, let us note that the joint $q\left(x_{1:T}\right)$ factorizes over instantaneous transition density (Chapman–Kolmogorov equation):

(40) $$q\left(x_{1:T}\right)=q\left(x_{1}\right)\overset{T}{\underset{t=2}{\prod }}q\left(x_{t}|x_{t-1}\right)=q\left(x_{1}\right)\frac{\overset{T}{\underset{t=2}{\prod }}q\left(x_{t},x_{t-1}\right)}{\overset{T}{\underset{t=2}{\prod }}q\left(x_{t-1}\right)}\text{.}$$

Therefore, its entropy decomposes into:

(41) $$S\left(q\left(x_{1:T}\right)\right)=-\overset{T}{\underset{t=2}{\sum }}\int \ln q\left(x_{t}|x_{t-1}\right)dq\left(x_{t}|x_{t-1}\right)+\overset{T-1}{\underset{t=2}{\sum }}\int \ln q\left(x_{t}\right)dq\left(x_{t}\right)=\frac{nT}{2}\left(\ln2\pi +1\right)+\frac{1}{2}\ln\left|\Psi_{1,1}\right|+\frac{1}{2}\overset{T}{\underset{t=1}{\sum }}\left(\ln\left|\begin{array}{cc} \Psi_{t,t} & \Psi_{t,t+1}\\ \Psi_{t,t+1}^{T} & \Psi_{t+1,t+1} \end{array}\right|-\ln\left|\Psi_{t,t}\right|\right)$$

where the matrix determinants are evaluated during the backward pass (when forming the αβ-messages) and the posterior lagged covariance is given by Eq. (38).

#### 3.2.5 Predictive and sojourn densities

Having identified the model, one may want to derive predictions about the evolution of the system. This requires the computation of a predictive density; i.e. the propagation of the posterior density over the hidden-states from the last observation. The predictive density can be accessed through the Chapman–Kolmogorov equation (Eq. (17)). However, the requisite integrals do not have an analytical solution. To finesse this problem we can extend our VB-Laplace approach to derive an approximation to the predictive density:

(42) $$\alpha_{t}^{*}\left(x_{t}|y_{1:T}\right)\propto \int \cdots \int q\left(x_{T}|y_{1:T}\right)\overset{t}{\underset{k=T+1}{\prod }}\exp{\langle \ln p\left(x_{t}|x_{t-1},\theta ,\alpha \right)\rangle }_{q\left(\theta \right)q\left(\alpha \right)}dx_{t-1}\propto \int \alpha_{t-1}^{*}\left(x_{t-1}|y_{1:T}\right)\exp{\langle \ln p\left(x_{t}|x_{t-1},\theta ,\alpha \right)\rangle }_{q\left(\theta \right)q\left(\alpha \right)}dx_{t-1}\approx N\left(\overset{*}{\underset{t}{m}},R_{t|T}\right)$$

for any t≥T+1. Here, the last line motivates a recursive Laplace approximation to the predictive density. As above, this is used to form a propagation equation for the mean and covariance of the approximate predictive density:

(43) $$m_{t}^{*}=f\left(m_{t-1}^{*},\mu_{\theta }\right)-{\overset{ˆ}{\alpha }}^{2}R_{t|T}{\frac{\partial f}{\partial x}}^{T}B_{t-1}^{-1}\frac{\partial^{2}\overset{˜}{f}}{\partial x\partial \theta }\left(I_{n}⊗\Sigma_{\theta }\right)\;\frac{\partial \overset{˜}{f}}{\partial \theta }$$

$$R_{t|T}={\overset{ˆ}{\alpha }}^{-1}{\left(I-\overset{ˆ}{\alpha }{\frac{\partial f}{\partial x}}^{T}B_{t-1}^{-1}\frac{\partial f}{\partial x}\right)}^{-1}$$

$$B_{t-1}=R_{t-1|T}^{-1}+\overset{ˆ}{\alpha }\left(\frac{\partial f}{\partial x}{\frac{\partial f}{\partial x}}^{T}+\frac{\partial^{2}\overset{˜}{f}}{\partial x\partial \theta }\left(I_{n}⊗\Sigma_{\theta }\right){\frac{\partial^{2}\overset{˜}{f}}{\partial x\partial \theta }}^{T}\right)\text{.}$$

Eq. (43) is used recursively in time to yield a Laplace approximation to the predictive density over hidden-states in the future. Similarly, we can derive an approximate predictive density for the data:

(44) $$\beta_{t}^{*}\left(y_{t}|y_{1:T}\right)\propto \int \alpha_{t}^{*}\left(x_{t}|y_{1:T}\right)\exp{\langle \ln p\left(y_{t}|x_{t},\varphi ,\sigma \right)\rangle }_{q\left(\varphi \right)q\left(\sigma \right)}dx_{t}\approx N\left(\overset{*}{\underset{t}{n}},Q_{t|T}\right)$$

which leads to the following moment propagation equations:

(45) $$n_{t}^{*}=g\left(n_{t-1}^{*},\mu_{\varphi }\right)-{\overset{ˆ}{\sigma }}^{2}Q_{t|T}{\frac{\partial g}{\partial x}}^{T}C_{t-1}^{-1}\frac{\partial^{2}\overset{˜}{g}}{\partial x\partial \varphi }\left(I_{n}⊗\Sigma_{\varphi }\right)\;\frac{\partial \overset{˜}{g}}{\partial \varphi }$$

$$Q_{t|T}={\overset{ˆ}{\sigma }}^{-1}{\left(I-\overset{ˆ}{\sigma }{\frac{\partial g}{\partial x}}^{T}C_{t-1}^{-1}\frac{\partial g}{\partial x}\right)}^{-1}$$

$$C_{t-1}=R_{t|T}^{-1}+\overset{ˆ}{\sigma }\left(\frac{\partial g}{\partial x}{\frac{\partial g}{\partial x}}^{T}+\frac{\partial^{2}\overset{˜}{g}}{\partial x\partial \varphi }\left(I_{n}⊗\Sigma_{\varphi }\right){\frac{\partial^{2}\overset{˜}{g}}{\partial x\partial \varphi }}^{T}\right)\text{.}$$

These equations are very similar to the predictive step of the forward pass of the VB-Laplace Kalman filter (Eq. (29)). They can be used for time-series prediction on hidden-states and measurements by iterating from t=T+1 to t=τ.

From the approximate predictive densities we can derive the approximate sojourn distribution over both state and measurement spaces. By definition, the sojourn distribution is the stationary density of the Markov chain, i.e. it is invariant under the transition density:

(46) $$p_{\infty }\left(x_{t}|m\right)=p_{\infty }\left(x_{t+1}|m\right)=\int p\left(x_{t+1}|x_{t},m\right)p_{\infty }\left(x_{t}|m\right)dx_{t}\text{.}$$

Estimating the sojourn density from partial observations of the system is a difficult inferential problem (see e.g. [42]). Here, we relate the sojourn distribution to the predictive density via the ergodic decomposition theorem [29]:

(47) $$p_{\infty }\left(x|m\right)=\underset{\tau \to \infty }{\lim}\;\frac{1}{\tau -T}\overset{\tau -1}{\underset{t=T}{\sum }}p\left(x_{t}|x_{0},m\right)\approx \frac{1}{\tau -T}\overset{\tau -1}{\underset{t=T}{\sum }}\overset{*}{\underset{t}{\alpha }}\left(x_{t}|y_{1:T}\right)$$

where τ−T is the number of predicted time steps and $\alpha_{t}^{*}\left(x_{t}|y_{1:T}\right)$ is the Laplace approximation of the predictive density at time t≥T+1 (Eqs. (42) and (43)). Eq. (47) subsumes three approximations: (i) the system is ergodic, (ii) a truncation of the infinite series of the ergodic decomposition theorem and (iii) a Laplace approximation to the predictive density. Effectively, Eq. (47) represents a mixture of Gaussian densities approximation to the sojourn distribution. It is straightforward to show that the analogous sojourn distribution in measurement space is given by:

(48) $$p_{\infty }\left(y|m\right)\approx \frac{1}{\tau -T}\overset{\tau -1}{\underset{t=T}{\sum }}\beta_{t}^{*}\left(y_{t}|y_{1:T}\right)$$

where $\beta_{t}^{*}\left(y_{t}|y_{1:T}\right)$ is the Laplace approximation to the measurement predictive density at time t≥T+1 (Eqs. (44) and (45)).

## 4 Evaluations of the VB-Laplace scheme

In this section, we try to establish the validity and accuracy of the VB-Laplace scheme using four complementary approaches:

• Comparative evaluations with the extended Kalman filter (EKF): We compared the estimation error of the VB-Laplace and EKF estimators in terms of estimation efficiency, when applied to systems with nonlinear evolution and observation functions.
• Bayesian model comparison: The application of the proposed scheme may include the identification of different forms or structures of state-space models subtending observed data. We therefore asked whether models whose structure could have generated the data are *a posteriori* more plausible than models that could not. To address this question we used the free-energy as a bound approximation to the log-model-evidence to compute an approximate posterior density on model space.
• Quantitative evaluation of asymptotic efficiency: Since our VB-Laplace approach provides us with an approximate posterior density, we assessed whether the VB estimator becomes optimal with large sample size.
• Assessment of time-series prediction: We explored the potential advantages and caveats in using the VB-Laplace approach for time-series prediction.

These analyses were applied to three well-known low-dimensional nonlinear stochastic systems; a double-well potential, Lorenz attractor and van der Pol oscillator. The dynamical behaviours of these systems cover diverse but important phenomena, ranging from limit cycles to strange attractors. These systems are described qualitatively below and their equations of motion are given in Table 1.

After having reviewed the dynamical properties of these systems, we will summarize the Bayesian decision theory used to quantify the performance of the method. Finally, we describe the Monte Carlo simulations used to compare VB-Laplace to the standard EKF, perform model comparison, assess asymptotic efficiency and characterise the prediction capabilities of VB-Laplace approach.

### 4.1 Simulated systems

#### 4.1.1 Double-well

The double-well potential system models a dissipative system, whose potential energy is a quadratic (double-well) function of position. As a consequence, the system is bistable with two basins of attraction to two stable fixed points, $\left(0,\theta_{1}\right)$ and $\left(0,\theta_{2}\right)$. In its deterministic variant, the system ends up spiralling around one or the other attractors, depending on its initial conditions and the magnitude of a damping force or dissipative term. Because we consider state-noise, the stochastic DCM can switch (tunnel) from one basin to the other, which leads to itinerant behaviour; this is why the double-well system can be used to model bistable perception [43].

**Fig. 2:**
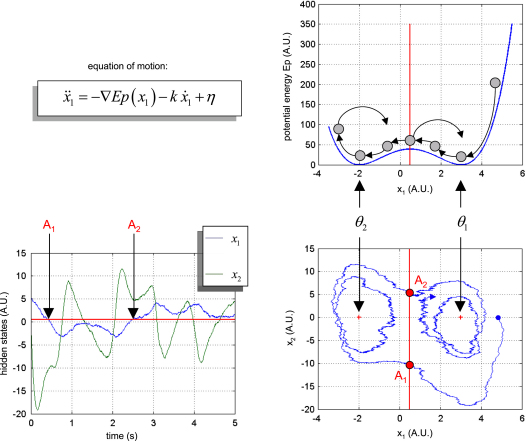
Double-well potential stochastic system: The double-well potential (as a function of position) and an example of a path (as a function of time in state-space) are shown. The system is bistable and its state-space exhibits two basin of attraction around two stable fixed points, $\left(0,\theta_{1}\right)$ and $\left(0,\theta_{2}\right)$. State-noise allows the state to “tunnel” from one basin to the other (see transition points $A_{1}$ and $A_{2}$), leading to itinerant dynamics.

Fig. 2 shows the double-well potential and a sample path of the system (as a function of time in state-space; $T=5\times 10^{3}$). In this example, the evolution parameters were $\theta ={\left(3,-2,3/2\right)}^{T}$, the precision of state-noise was $\alpha =10^{3}$ and the initial conditions were picked at random. The path shows two jumps over the potential barrier (points $A_{1}$ and $A_{2}$), the first being due primarily to kinetic energy ($A_{1}$), and the second to state-noise ($A_{2}$). Between these two, the path spirals around the stable attractors.

#### 4.1.2 Lorenz attractor

The Lorenz attractor was originally proposed as a simplified version of the Navier–Stokes equations, in the context of meteorological fluid dynamics [44]. The Lorenz attractor models the autonomous formation of convection cells, whose dynamics are parameterized using three parameters; $\theta_{1}$: the Rayleigh number, which characterizes the fluid viscosity, $\theta_{2}$: the Prandtl number which measures the efficacy of heat transport through the boundary layer and $\theta_{3}$: a dissipative coefficient. When the Rayleigh number is bigger than one, the system has two symmetrical fixed points $\left(\pm \sqrt{\theta_{3}\left(\theta_{1}-1\right)},\pm \sqrt{\theta_{3}\left(\theta_{1}-1\right)},\theta_{1}-1\right)$, which act as a pair of local attractors. For certain parameter values; e.g., $\theta ={\left(28,10,8/3\right)}^{T}$, the Lorenz attractor exhibits chaotic behaviour on a butterfly-shaped strange attractor. For almost any initial conditions (other than the fixed points), the trajectory unfolds on the attractor. The path begins spiralling onto one wing and then jumps to the other and back in a chaotic way. The stochastic variant of the Lorenz system possesses more than one random attractor. However, with the parameters above, the sojourn distribution settles around the deterministic strange attractor [45].

**Fig. 3:**
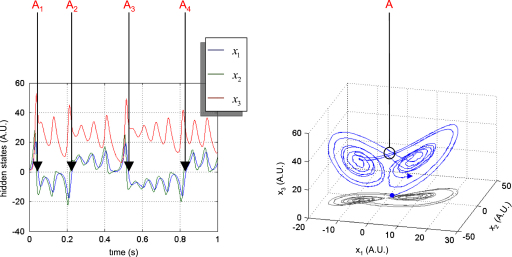
Lorenz attractor: A sample path of the Lorenz system is shown as a function of time (left) and in state-space (right). The Lorenz attractor is a butterfly-shaped strange attractor: the path begins spiralling onto one wing and then jumps onto to the other and so forth, in a chaotic way. Points $A_{1}$, $A_{2}$, $A_{3}$ and $A_{4}$ are transition points from one wing to the other.

Fig. 3 shows a sample path of the Lorenz system ($T=5\times 10^{2}$). In this example, the evolution parameters were set as above, the precision of state-noise was $\alpha =10^{2}$ and the initial conditions were picked at random. The path shows four jumps from one wing to the other.

#### 4.1.3 van der Pol oscillator

**Fig. 4:**
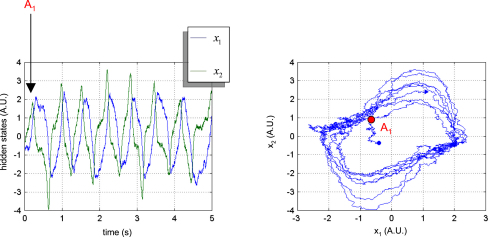
van der Pol oscillator: A sample path of the van der Pol oscillator (as a function of time and in state-space) is shown. In this example, the deterministic variant of the system is stable and possesses a limit cycle. The sample path $\left(T=5\times 10^{3}\right)$ shows four periods of the quasi-limit cycle, following a short transient (point $A_{1}$) converging towards the attractor manifold.

The van der Pol oscillator has been used as the basis for neuronal action potential models [46,47]. It is a non-conservative oscillator with nonlinear damping parameterized by a single parameter, $\theta_{1}$. It is a stable system for all initial conditions and dampening parameter. When $\theta_{1}$ is positive, the system enters a limit cycle. Fig. 4 shows a sample path ($T=5\times 10^{3}$) of the van der Pol oscillator. In this example, the evolution parameter was θ=1, the precision of state-noise was $\alpha =10^{3}$ and the initial conditions were picked at random. The path exhibits four periods of a quasi-limit cycle after a short transient (point $A_{1}$).

### 4.2 Estimation loss and statistical efficiency

The statistical efficiency of an estimator is a decision theoretic measure of accuracy [34]. Given the true parameters of the generative model and their estimator, we can evaluate the squared error loss SEL(ϑ) with:

(49) $$SEL\left(\vartheta \right)=\underset{i}{\sum }{\left(\vartheta_{i}-{\overset{ˆ}{\vartheta }}_{i}\right)}^{2}$$

where ${\overset{ˆ}{\vartheta }}_{i}$ is the ith element of the estimator of $\vartheta \in \left\{x_{1:T},x_{0},\theta ,\alpha ,\sigma \right\}$. The SEL is a standard estimation error measure, whose *a posteriori* expectation is minimized by the posterior mean. In Bayesian decision theoretic terms, this means that an estimator based on the posterior mean; $\overset{ˆ}{\vartheta }={\langle \vartheta \rangle }_{q}$ is optimal with respect to squared error loss.

It can be shown that the expected SEL under the joint density p(y,ϑ|m) is bounded by the Bayesian Fisher information:

(50) $${\langle SEL\left(\vartheta \right)\rangle }_{p\left(y,\vartheta |m\right)}\geq {\left({\langle \frac{\partial^{2}}{\partial \vartheta^{2}}\ln p\left(y,\vartheta |m\right)\rangle }_{p\left(\vartheta ,y|m\right)}\right)}^{-1}\text{.}$$

Eq. (50) gives the so-called Bayesian Cramer–Rao bound, which quantifies the minimum average SEL, under the generative model m[48]. By definition, the proximity to the Cramer–Rao bound measures the efficiency of an approximate Bayesian estimator. The efficiency of the method is related to the amount of available information, which, when the observation function is the identity mapping ($g\left(x\right)=I_{n}$), is proportional to the sample size T. In this case, asymptotic efficiency is achieved whenever estimators attain the Cramer–Rao bound when T→∞.

In addition to efficiency, we also evaluated the approximate posterior confidence intervals. As noted above, under the Laplace assumption, this reduces to assessing the accuracy of the posterior covariance. In decision theoretic terms, confidence interval evaluation, under the Laplace approximation, is equivalent to squared error loss estimation, since:

(51) $$EL\left(q\right)={\langle SEL\left(\vartheta \right)\rangle }_{q\left(\vartheta \right)}=\mathrm{tr}\left(\Sigma_{\vartheta }\right)$$

where the *a posteriori* expected loss EL(q) is the Bayesian estimator of SEL. EL(q) thus provides a self-consistency measure that is related to confidence intervals (see [34]).

### 4.3 Comparing VB-Laplace and EKF

The EKF provides an approximation to the posterior density on the hidden-states of the state-space model given in Eq. (11). The standard variant of the EKF uses a forward pass, comprising a prediction and an update step (see e.g. [16]):

(52) $$\text{Prediction step:}\;\left\{ \begin{array}{c} m_{t}^{*}=f\left(m_{t-1}\right)\\ R_{t|t-1}=\alpha I+{\frac{\partial f}{\partial x}}^{T}R_{t-1|t-1}\frac{\partial f}{\partial x} \end{array}\right.$$

$$\text{Update step:}\;\left\{ \begin{array}{c} m_{t}=m_{t}^{*}+\sigma R_{t|t}\frac{\partial g}{\partial x}\left(y_{t}-g\left(m_{t}^{*}\right)\right)\\ R_{t|t}={\left(R_{t|t-1}^{-1}+\sigma \frac{\partial g}{\partial x}{\frac{\partial g}{\partial x}}^{T}\right)}^{-1}\text{.} \end{array}\right.$$

These two steps are iterated from t=1 to t=T. It is well known that both model misspecification (e.g. using incorrect parameters and hyperparameters) and local linearization can introduce biases and errors in the covariance calculations that degrade EKF performance [49].

We conducted a series of fifty Monte Carlo simulations for each dynamical system. The observation function for all three systems was taken to be the following sigmoid mapping:

(53) $$g\left(x\right)=\frac{G_{0}}{1+\exp\left(-bx\right)}$$

where the constants $\left(G_{0},b\right)$ were chosen to ensure changes in hidden-states were of sufficient amplitude to cause nonlinear effects (i.e. saturation) in measurement space. Table 2 shows the different simulation and prior parameters for the dynamical systems we examined.

Note that the standard EKF cannot estimate parameters or hyperparameters. Therefore, we have used two EKF versions: EKF1 used the prior means of the parameters (${\langle \vartheta \rangle }_{p\left(\vartheta \right)}$), and EKF2 uses their posterior mean from the VB-Laplace algorithm (${\langle \vartheta \rangle }_{q\left(\vartheta \right)}$).

**Fig. 5:**
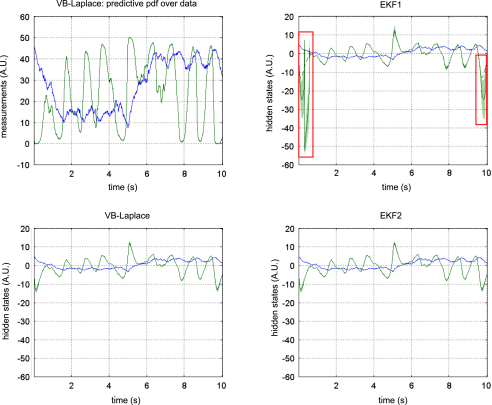
Comparison between the VB-Laplace and the EKF approaches: a double-well potential example: The figure depicts the estimated hidden-states of a simulated Double-well system as given respectively by the VB-Laplace and the EKF methods. Top-left: first- (solid line) and second-order (shaded area) moments of the VB-Laplace approximate predictive density over observations, and simulated data (dashed line — here superimposed). Bottom-left: first- (solid line) and second-order (shaded area) moments of the VB-Laplace approximate posterior density over hidden-states, and simulated hidden-states (dashed line). Top-right: first- (solid line) and second-order (shaded area) moments of the EKF1 approximate posterior density over hidden-states, and simulated hidden-states (dashed line). Top-right: first- (solid line) and second-order (shaded area) moments of the EKF2 approximate posterior density over hidden-states, and simulated hidden-states (dashed line). The second-order moment is represented using the 90% posterior confidence interval (shaded area). Red boxes highlight typical estimation instabilities of the EKF, which are not evidenced by the VB-Laplace approach. Note that when the first-order moment matches the simulated variable, the dashed line is hidden by the solid line.

**Fig. 6:**
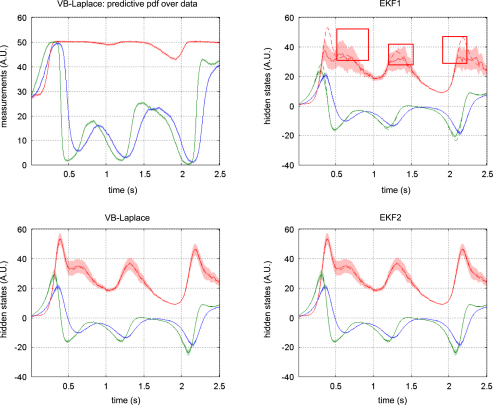
Comparison between the VB-Laplace and the EKF approaches: a Lorenz attractor example: This figure uses the same format as Fig. 5.

**Fig. 7:**
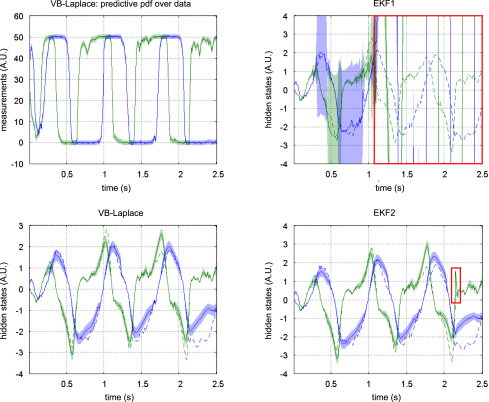
Comparison between the VB-Laplace and the EKF approaches: a van der Pol oscillator example: This figure uses the same format as Fig. 5.

Figs. 5–7 show the results of the comparative evaluations of VB-Laplace, EKF1 and EKF2, where these and subsequent figures use the same format:

• Top-left: first- and second-order moments of the approximate predictive density on the observations (and simulated data) as given by VB-Laplace.
• Bottom-left: first- and second-order moments of the approximate posterior density on the hidden-states (and simulated hidden-states) as given by VB-Laplace.
• Top-right: first- and second-order moments of the approximate posterior density on the hidden-states (and simulated hidden-states) as given by EKF1.
• Bottom-right: first- and second-order moments of the approximate posterior density on the hidden-states (and simulated hidden-states) as given by the EKF2.

It can be seen that despite the nonlinear observation and evolution functions, both VB-Laplace and EKF2 estimate the hidden-states accurately. Furthermore, they both provide reliable posterior confidence intervals. This is not the case for the EKF1, which, in these examples, exhibits significant estimation errors.

**Fig. 8:**
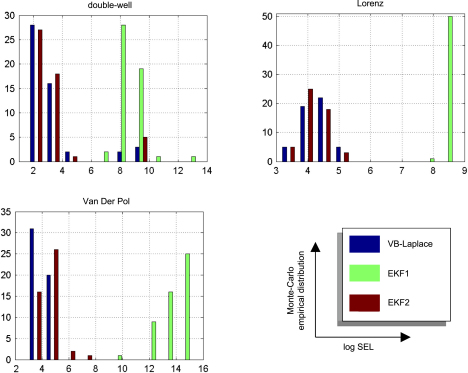
Monte Carlo comparison between the VB-Laplace and the EKF approaches: The empirical Monte Carlo distributions of the SEL score (on a logarithmic scale) for all methods (VB-Laplace, EKF1 and EKF2), as a function of the simulated system (top-left: double-well, top-right: Lorenz, bottom-left: van der Pol).

We computed the SEL score on the hidden-states for the three approaches. The Monte Carlo distributions of this score are given in Fig. 8. There was always a significant difference (one-sample paired t-test, 5% confidence level, df = 49) between the VB-Laplace and the EKF1 approaches, with the VB-Laplace method exhibiting greater efficiency. This difference is greatest for the van der Pol system, in which the nonlinearity in the observation function was the strongest. There was a (less) significant difference between the VB-Laplace and the EKF2 approaches for the Lorenz and the van der Pol systems; VB-Laplace is more (respectively less) efficient than the EKF2 when applied to the van der Pol (respectively Lorenz) system. Table 3 summarizes these results. It is also worth reporting that 11% of the Monte Carlo simulations led to numerical divergences of the EKF2 algorithm for the van der Pol system (these were not used for when computing the paired t-test).

To summarize, the EKF seems sensitive to model misspecification. This is why the EKF1 (relying on prior means) performs badly when compared to the EKF2 (relying on the VB-Laplace posterior means). This is not the case for the VB-Laplace approach, which seems more robust to model misspecification. In addition, the EKF seems very sensitive to noise in presence of strong nonlinearity (cf. numerical divergence of EKF2 for the van der Pol system). It could be argued that the good estimation performances achieved by EKF2 are inherited from the VB-Laplace through the posterior parameter estimates and implicit learning of the structure of the hidden stochastic systems.

### 4.4 Assessing VB-Laplace model comparison

Here, we asked whether one can identify the structure of the hidden stochastic system using Bayesian model comparison based on the free-energy. We assessed whether models whose structure could have generated the data are *a posteriori* more plausible than models that could not. To do this, we conducted another 50 Monte Carlo simulations for each of the three systems. For each of these simulations, we compared two classes of models: the model used to generate the simulated data (referred to as the “true” model) and a so-called “generic” model, which was the same as the true model except for the form of the evolution function:

(54) f(x,θ)=Ax+BQ(x)

Q(x)={xixj}i=1,…,nj≥i

where the elements of the matrices θ={A,B} were unknown and estimated using VB-Laplace. The number of evolution parameters θ depends on the number of hidden-states: $n_{\theta }=n\left(2n+\frac{1}{2}n!/\left(n-2\right)!\right)$. This evolution function can be regarded as a second-order Taylor expansion of the equations of motion; f(x). This means that the generic model recover the dynamical structure of the Lorenz system, which is a generic model with the following parameters:

(55) $$A=\left[\begin{array}{ccc} -10 & 10 & 0\\ 28 & -1 & 0\\ 0 & 0 & 8/3 \end{array}\right]\text{,}\;B=\left[\begin{array}{cccccc} 0 & 0 & 0 & 0 & 0 & 0\\ 0 & 0 & -1 & 0 & 0 & 0\\ 0 & 1 & 0 & 0 & 0 & 0 \end{array}\right]\text{.}$$

However, the generic model cannot capture the dynamical structure of the van Der Pol and double-well systems (cf. Table 1). The specifications of the generative models are identical to those given in Table 2, except for the “generic” generative model, for which the priors on the evolution parameters are given in Table 4.

**Fig. 9:**
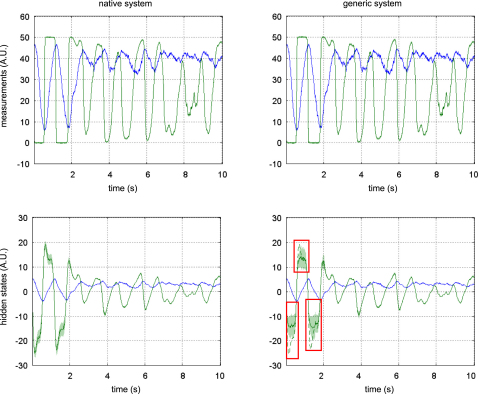
Comparison between the VB-Laplace inversion of the true model and of the generic model: a double-well potential example: This figure shows the VB-Laplace estimator of the hidden-states of a simulated Double-well system under both the true and generic models. Top-left: first- (solid line) and second-order (shaded area) moments of the VB-Laplace approximate predictive density over observations, and simulated data (dashed line), under the true model. Bottom-left: first- (solid line) and second-order (shaded area) moments of the VB-Laplace approximate posterior density over hidden-states, and simulated hidden-states (dashed line), under the true model. Top-right: first- (solid line) and second-order (shaded area) moments of the VB-Laplace approximate predictive density over observations, and simulated data (dashed line), under the generic model. Bottom-left: first- (solid line) and second-order (shaded area) moments of the VB-Laplace approximate posterior density over hidden-states, and simulated hidden-states (dashed line), under the generic model. The second-order moment is represented using the 90% posterior confidence interval (shaded area). Red boxes highlight significant estimation errors of the VB-Laplace approach, under the generic model.

**Fig. 10:**
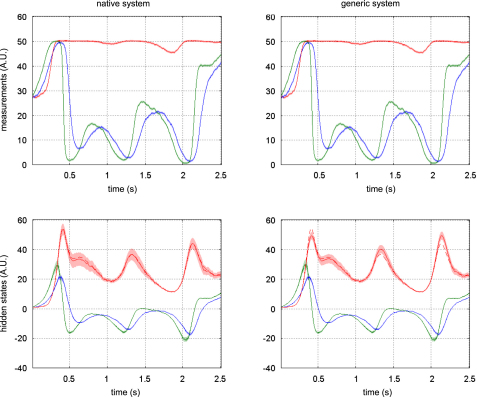
Comparison between the VB-Laplace inversion of the true model and of the generic model: a Lorenz attractor example: This figure uses the same format as Fig. 9.

**Fig. 11:**
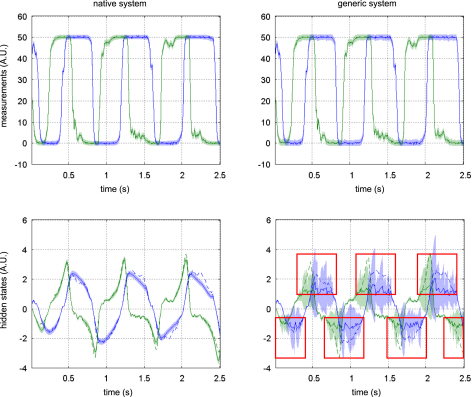
Comparison between the VB-Laplace inversion of the true model and of the generic model: a van der Pol oscillator example: This figure uses the same format as Fig. 9.

Figs. 9–11 compare the respective VB-Laplace inversion of the true and the generic generative models; specifically

• Top-left: first- and second-order moments of the approximate predictive density on the observations (and simulated data) under the true model.
• Bottom-left: first- and second-order moments of the approximate posterior density on the hidden-states (and simulated hidden-states) under the true model.
• Top-right: first- and second-order moments of the approximate predictive density on the observations (and the simulated data) under the generic model.
• Bottom-right: first- and second-order moments of the approximate posterior density on the hidden-states (and simulated hidden-states) under the generic model.

It can be seen from these figures that the Lorenz system’s hidden-states are estimated well under both the true and generic models. This is not the case for the van der Pol and the double-well systems, for which the estimation of the hidden-states under the generic model deviates significantly from the simulated time-series. Note also that the posterior confidence intervals reflect the mismatch between the simulated and estimated hidden-states. This is most particularly prominent for the van der Pol system (Fig. 11), where the posterior variances increase enormously, whenever the observations fall on the nonlinear (saturation) domain of the sigmoid observation function. Nevertheless, for both true and generic models, the data were predicted almost perfectly for all three systems: the measured data always lie within the confidence intervals of the approximate predictive densities.

**Fig. 12:**
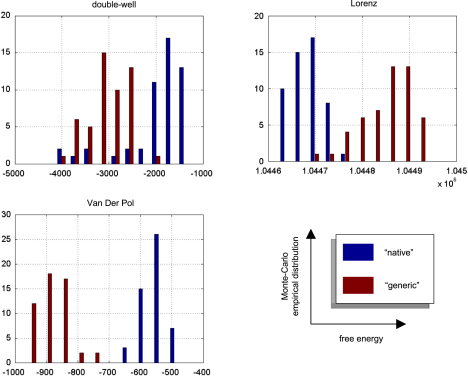
Monte Carlo assessment of the VB-Laplace model comparison capabilities: The empirical Monte Carlo distributions of the free-energy are given for both models (true and generic), as a function of the simulated system (top-left: double-well, top-right: Lorenz, bottom-left: van der Pol).

The VB-Laplace approach provides us with the free-energy of the true and generic models for each Monte Carlo simulation. Its empirical Monte Carlo distribution for each class of systems is shown in Fig. 12. In addition, for each simulation, we computed the standard “goodness-of-fit” sum of squared error $SSE=\ln\sum_{t}{\left(y_{t}-{\overset{ˆ}{y}}_{t}\right)}^{2}$, which is the basis for any non-Bayesian statistical model comparison. Finally, we computed the estimation loss (SEL) on the hidden-states, which cannot be obtained in real applications. These performance measures allowed us to test for significant differences between the true and generic models in terms of their free-energy, SSE and SEL. The results are summarized in Table 5.

Unsurprisingly, the estimation loss (SEL) was always significantly smaller for the true model. This means that the hidden-states were always estimated more accurately under the true, relative to the generic model. More surprisingly (because the fits looked equally accurate), there was always a significant difference between the true and generic models, in terms of their goodness-of-fit (SSE). However had we based our model comparison on this index, we would have favoured the generic model over the true van der Pol system.

**Fig. 13:**
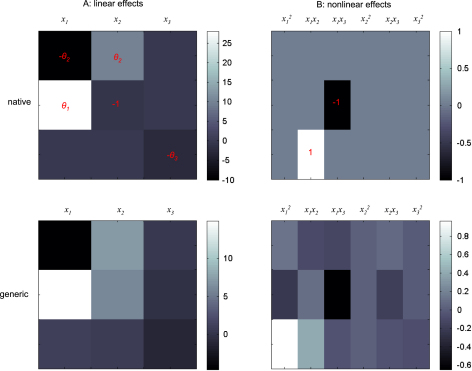
Comparison between the dynamical structure of the true Lorenz system and its VB-Laplace estimation under the generic model: The figure depicts the matrices A encoding linear effects (left) and B nonlinear effects (right) of the generic model. The top row shows the true A and B matrices of the Lorenz model, which can be expressed in the generic form. The bottom row shows the Monte Carlo average of the VB-Laplace estimator of the A and B matrices, under the generic model.

There was always a significant difference between the true and generic models in terms of free-energy. Model comparison based on the free-energy would have led us to select the true against the generic model for the Double-well and van der Pol — but not for the Lorenz system. This is what we predicted, because the generic model covers the dynamical structure of the Lorenz system. Fig. 13 shows the Monte Carlo average of the posterior means of both matrices A and B, given data generated by the Lorenz system. The inferred structure is very similar to the true system. Note however; (i) the global rescaling of the Monte Carlo average of the A matrix relative to its Lorenz analogue and (ii) the slight ambiguity regarding the contributions of the nonlinear $x_{1}^{2}$ and $x_{1}x_{2}$ effects on $x_{3}$. The global rescaling is due to the “minimum norm” priors imposed on the evolution parameters of the generic model. The fact that the nonlinear effects on $x_{3}$ are shared between the quadratic $x_{1}^{2}$ and $x_{1}x_{2}$ interaction terms is due to the strong correlation between the time-series of $x_{1}$ and $x_{2}$ (see e.g. Figs. 3, 6 and 10). We discuss the results of this model comparison below.

### 4.5 Assessing the asymptotic efficiency of the VB-Laplace approach

In this third set of simulations, we asked whether the VB-Laplace estimation accuracy is close to optimal and assessed the quality of the posterior confidence intervals, when the sample size becomes large. In other words, we wanted to understand the influence of sample size on the estimation capabilities of the method. To do this, we used the simplest observation function; the identity mapping: $g\left(x\right)=I_{n}$ and varied sample size. This means we could evaluate the behaviour of the measured squared error loss SEL(T) as a function of sample size T, for each of the three nonlinear stochastic systems above.

We conducted a series of fifty Monte Carlo simulations for seven sample sizes (T∈[5;10;50;100;500;1000;5000]) and for each dynamical system. Table 3 shows the simulated and prior parameters used.

We applied the VB-Laplace scheme to each of these 1050 simulations. We then calculated the squared error loss (SEL) and expected loss (EL)^8^ from the ensuing approximated posterior densities.

Sampling the empirical Monte Carlo distributions of both these evaluation measures allowed us to approximate their expectation under the marginal likelihood. Therefore, characterising the behaviour of Monte Carlo average SEL as a function of the sample size T provides a numerical assessment of asymptotic efficiency. Furthermore, comparing the Monte Carlo average SEL and Monte Carlo average EL furnishes a quantitative validation of the posterior confidence intervals.

**Fig. 14:**
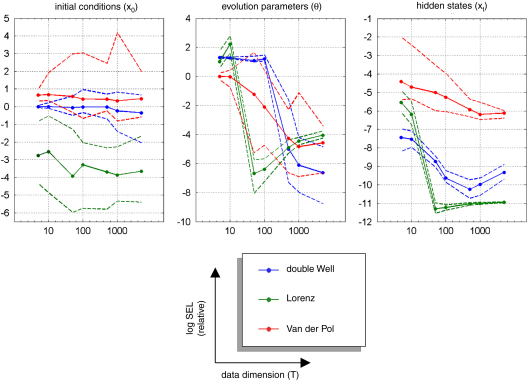
Monte Carlo evaluation of estimation accuracy: states and parameters: The solid line (respectively the dashed line) plots the Monte Carlo 50% percentile (respectively the Monte Carlo 10% and 90% percentiles) of the log relative SEL for the initial conditions, evolution parameters and hidden-states, for each dynamical system, as a function of the number of time-samples T.

**Fig. 15:**
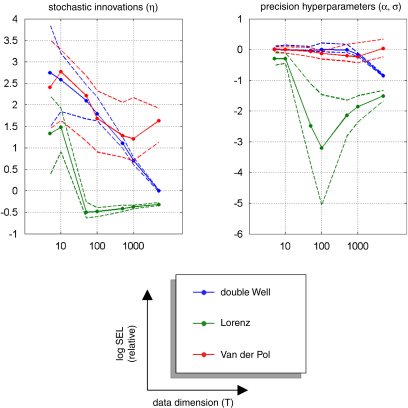
Monte Carlo evaluation of estimation accuracy: state-noise and precision hyperparameters: This figure uses the same format as Fig. 14.

Fig. 14 (resp. Fig. 15) shows the Monte Carlo distributions (10%, 50% and 90% percentiles) of the relative squared error for the initial conditions, evolution parameters and hidden-states (resp. the estimated state-noise ${\overset{ˆ}{\eta }}_{0:T-1}$ and the precision hyperparameters). Except for the initial conditions, all the VB-Laplace estimators show a jump around T=100; above which the squared error loss seems to asymptote. Moreover, the VB-Laplace estimators of both evolution parameters θ and hidden-states $x_{1:T}$ exhibit a significant (quasi-monotonic) variation with T (see Fig. 14).^9^ On average, and within the range of T we considered, the squared root loss seems to be inversely related to the sample size T:

(56) $$\frac{SEL\left(\min\left(T\right)\right)}{SEL\left(\max\left(T\right)\right)}\propto \frac{\max\left(T\right)}{\min\left(T\right)}\text{.}$$

This would be expected when estimating the parameters of a linear model, since (under a linear model) the Cramer–Rao bound is:

(57) $${\langle SEL\left(\vartheta \right)\rangle }_{p\left(\vartheta ,y|m\right)}=\mathrm{trace}\left[\Sigma_{\vartheta }\right]\;\propto df^{-1}$$

where df enumerates the degrees of freedom. However, we are dealing with nonlinear models, whose number of unknowns (the hidden-states) increases with sample size and for which no theoretical bound is available. Nevertheless, our Monte Carlo simulations suggest that Eq. (57) seems to be satisfied over the range of T considered. This result seems to indicate that the VB-Laplace estimator of both hidden-states and evolution parameters attains asymptotic efficiency.

Surprisingly, the estimation efficiency for the initial conditions $x_{0}$ does not seem to be affected by the sample size because it does not show significant variation within the range of T considered. This might be partially explained by the fact that the systems we are dealing with are close to ergodic. If the system is ergodic, then there is little information about the initial conditions at the end of the time-series. In this case, the approximate marginal posterior density of the initial conditions depends weakly on the sample size. This effect also interacts with the mean-field approximation: the derivation of the approximate posterior density of the initial conditions $q\left(x_{0}\right)$ depends primarily on that of the first hidden-state $q\left(x_{1}\right)$ through the message passing algorithm.^10^ Therefore, it should not matter whether we increase the sample size: the effective amount of available information for the initial conditions is approximately invariant. Lastly, we note a significant variation of the estimation efficiency for both the state-noise and the precision hyperparameters (except for the van der Pol case: see Fig. 9). This efficiency gain is qualitatively similar to that of evolution parameters and hidden-states, though to a lesser extent.

**Fig. 16:**
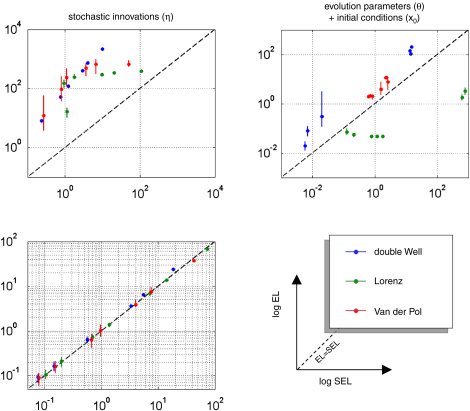
Monte Carlo evaluation of posterior confidence intervals: The three panels show the relationship between the Monte Carlo mean squared error loss (SEL) and its posterior expectation (EL=〈SEL〉) as log–log plots, for the three dynamical systems. Dots (respectively bars) show the Monte Carlo mean (respectively the 90% Monte Carlo confidence intervals) as a function of the sample size: T∈[5;10;50;100;500;1000;5000]. These are shown for state-noise (top-left), hidden-states (bottom-left), evolution parameters and initial conditions (top-right). The EL=SEL dashed line depicts perfect self-consistency; i.e. expected loss is equal to measured loss. The area above this diagonal corresponds to underconfidence, where expected loss is greater than measured loss.

Fig. 16 shows the VB-Laplace self-consistency measure, in terms of the quantitative relationship between the measured loss (SEL) and its posterior expectation (EL=〈SEL〉). To demonstrate the ability of the method to predict its own estimation error, we constructed log–log scatter plots of the posterior loss versus measured loss (having pooled over simulation) for hidden-states ($x_{1:T}$), parameters (θ and $x_{0}$) and state-noise ($\eta_{0:T-1}$). The hidden-states show a nearly one-to-one mapping between measured and expected loss, which is due to the fact that the hidden-states populated the lowest level in the hierarchical model. As a consequence, the VB-Laplace approximation to their posterior density does not suffer from having to integrate over intermediate levels. Both the evolution parameters and initial conditions show a close relationship between measured and expected loss. Nevertheless, it can be seen from Fig. 16 that the VB-Laplace estimates of the evolution parameters for the double-well and the van der Pol system are slightly underconfident. This underconfidence is also observed for the state-noise precision. This might partially be due to a slight but systematic underestimation of the state-noise precision hyperparameter α.This pessimistic VB-Laplace estimation of the squared error loss (SEL) would lead to conservative posterior confidence intervals.

However, note that this underconfidence is not observed for the Lorenz parameters, whose VB-Laplace estimation appears to be slightly overconfident (shrinked posterior confidence intervals). This is important, since this means that the bias of posterior confidence interval VB-Laplace estimation depends upon the system to be inverted. These underconfidence/overconfidence effects are discussed in details below (see discussion section “*On asymptotic efficiency*”).

### 4.6 Assessing prediction ability

**Fig. 17:**
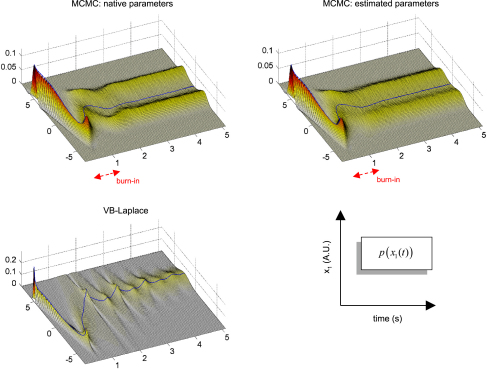
Short-term predictive power of the VB-Laplace approach: the double-well system: The figure compares the VB-Laplace approximation to the predictive density over hidden-states (bottom) with that obtained from MCMC sampling (top). Only the predictive density over the first hidden-state ($x_{1}$) is shown. Top-left: MCMC predictive density using the true parameters. Top-right: MCMC predictive density using the VB-Laplace estimates. The red arrows depict the burn-in period (before entering a quasi-stationary bimodal state).

**Fig. 18:**
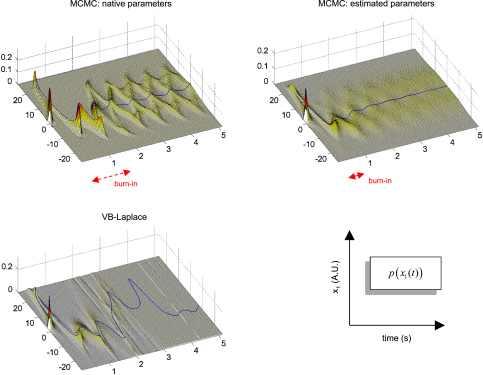
Short-term predictive power of the VB-Laplace approach: the Lorenz system: This figure uses the same format as Fig. 17.

**Fig. 19:**
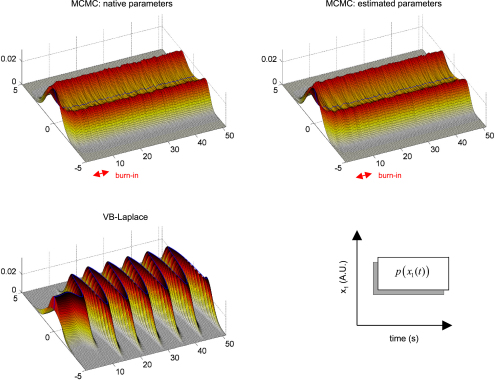
Short-term predictive power of the VB-Laplace approach: the Lorenz system: This figure uses the same format as Figs. 17 and 18.

Finally, we assessed the quality of the predictive and sojourn densities. Figs. 17–19 show the approximate predictive densities over the hidden-states ($\alpha_{t}^{*}\left(x_{t}\right)$), as given by VB-Laplace and a standard Monte Carlo Markov Chain (MCMC) sampling technique [35], for each of the three dynamical systems. Specifically:

• Top-left: MCMC predictive density using the true parameters.
• Top-right: MCMC predictive density using the parameters and hyperparameters estimated by the VB-Laplace approach.
• Bottom-left: VB-Laplace approximate predictive density using the parameters and hyperparameters estimated by VB-Laplace.

Note that we used the Monte Carlo averages of the VB-Laplace posterior densities parameters and hyperparameters from the first series of Monte Carlo simulations. After a “burn-in” period, the predictive density settles down into stationary (double-well and van der Pol) or cyclostationary^11^ (Lorenz) states that are multimodal.^12^

The double-well system (Fig. 17) exhibits a stationary bimodal density whose modes are centred on the two wells. Its burn-in period is similar for both MCMC estimates (ca. one second). The bimodality occurs because of diffusion over the barrier caused by state-noise. The Lorenz system (Fig. 18) shows a quasi-cyclostationary predictive density, after a burn-in period of about 1.5 s under the true parameters, and 0.8 s under their VB estimates. Note that due to the diffusive effect of state-noise, this quasi-cyclostationary density slowly converges to a stationary density (not shown). Within a cycle, each mode reproduces the trajectory of one oscillation around each wing of the Lorenz attractor. The bimodality of the Lorenz predictive density is very different in nature to that of the double-well system. First, there are periodic times at which the two modes co-occur, i.e. for which the predictive density can be considered as unimodal. This occurs approximately every 700 ms. At these times the states are close to the transition point $x_{1}=x_{2}=0$ between the two attractor wings. At this transition point, state-noise allows the system to switch to one or the other wing of the attractor. However, the trajectory between transition points is quasideterministic, i.e. it evolves in the neighbourhood of the deterministic orbit around the chosen wing. This is because the evolution function is dominated by the deterministic part of the evolution function. The van der Pol system (Fig. 19) shows a stationary bimodal density, after a burn-in period of about 1 s. The modes of the stationary density are centred on the extremal values of its deterministic variant (around $x_{1}=\pm 2$). Here again, the bimodality of the van der Pol predictive density is very different from the two other systems. The main effect of state-noise is to cause random jitter in the phase of the van der Pol oscillator. In addition, the system slows down when approaching extremal values. As a consequence, an ensemble of stochastic van der Pol oscillator will mostly populate the neighbourhoods of both the extremal values of the deterministic oscillator.

The stationarity in each of the three systems seems to be associated with ergodicity (at least for the first moment of the predictive density). Note that both the form of the stationary density and the burn-in period depends upon the structure of the dynamical system, and particularly on the state-noise precision hyperparameter. This latter dependence is expressed acutely in the Lorenz attractor (Fig. 18): the modes of the cyclostationary distribution under the true parameters and hyperparameters are wider than those under the VB estimates. Also, the burn-in period is much shorter under the VB estimates. This is due to the fact that the state-noise precision hyperparameter has been underestimated.

The VB-Laplace approximation to the predictive density cannot reproduce the multimodal structure of the predictive density (Figs. 17, 18 and 19). However, it is a good approximation to the true predictive density during the burn-in period. It can be seen from Figs 17, 18 and 19 that the burn-in MCMC unimodal predictive density is very similar to its VB-Laplace approximation, except for the slight overconfidence problem. Note also the drop in the precision of the VB-Laplace approximate predictive density after the burn-in period, for both the double-well and the Lorenz system. This means that the VB-Laplace approach predicts its own inaccuracy, after the burn-in period. In summary, these results mean that, contrary to middle-term predictions, short-term predictions are not compromised by the Gaussian approximation to the predictive density. By short-term predictions, we mean predictions over the burn-in period. The accuracy of the VB-Laplace predictions shows a clear transition when the system actually becomes ergodic. When this is the case (middle-term), the VB-Laplace predictions become useless.

**Fig. 20:**
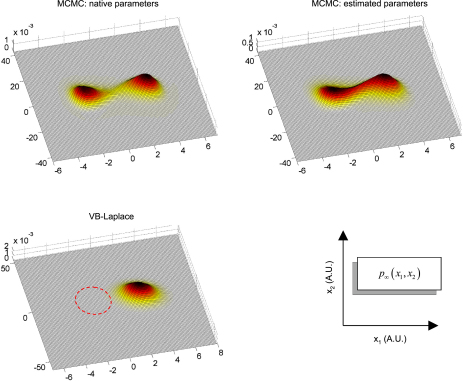
Long-term predictive power of the VB-Laplace approach: the double-well system: The figure compares the VB-Laplace approximation to the sojourn density over hidden-states (bottom) with that obtained from MCMC sampling (top). Top-left: MCMC predictive density using the true parameters. Top-right: MCMC predictive density using the VB-Laplace estimates. The red dashed circle depicts the position of the missing mode of the sojourn density.

**Fig. 21:**
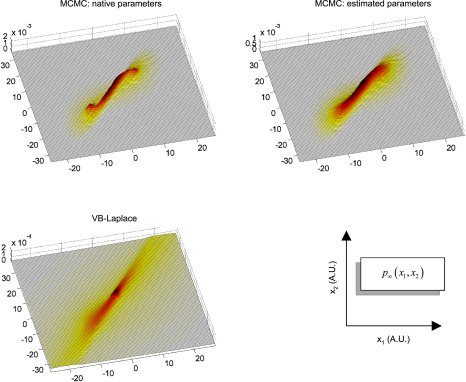
Long-term predictive power of the VB-Laplace approach: the Lorenz system: This figure uses the same format as Fig. 20. Note that the sojourn density has been marginalized over $x_{3}$ to give $p_{\infty }\left(x_{1},x_{2}\right)$.

**Fig. 22:**
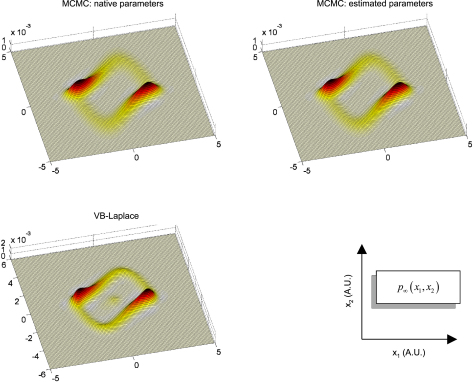
Long-term predictive power of the VB-Laplace approach: the van der Pol system: This figure uses the same format as Figs. 20 and 21.

Figs. 20–22 depict the sojourn distributions as given by VB-Laplace and Monte Carlo Markov Chain (MCMC) sampling, for each of the three dynamical systems. The MCMC sojourn density of the double-well system (Fig. 20) is composed of two (nearly Gaussian) modes, connected to each other by a “bridge”. The difference between the amplitudes of this bridge under the true parameters and under the VB estimates is again due to a slight underestimation of the state-noise precision hyperparameter. As can be seen from Fig. 20, the approximate sojourn distribution of the Double-Well system is far from perfect: one of the two modes (associated with the left potential well) is missing. This is due to the fact that the Gaussian approximation to the predictive density cannot account for stochastic phase transitions. This means that the prediction for this system will be biased by the initial conditions (last *a posteriori* inferred state), and will get worse with time. In contrast, Figs. 21 and 22 suggest a good agreement between VB-Laplace approximate and MCMC sampled sojourn distributions for the Lorenz and van der Pol systems. Qualitatively, their state-space maps seem to be recovered correctly, ensuring a robust long-term (average) prediction. Note that the lack of precision of the Lorenz VB-Laplace approximate sojourn density (Fig. 21) is mainly due to the underestimation of the state-noise precision hyperparameter, since the same “smoothing” effect is noticeable on the MCMC sojourn distribution under the VB hyperparameters. The structure of the van der Pol sojourn distribution is almost perfectly captured, except for a slight residual from the initial conditions (centred on the fixed point $x_{1}=x_{2}=0$).

Taken together, these preliminary results indicate that the long-term predictive power of the VB-Laplace scheme depends on the structure of the stochastic system to be predicted. This means that accuracy of the VB-Laplace long-term predictions might only hold for a certain class of stochastic nonlinear systems (see Section 5).

## 5 Discussion

We have proposed a variational Bayesian approach to the inversion and prediction of nonlinear stochastic dynamic models. This probabilistic technique yields (i) approximate posterior densities over hidden-states, parameters and hyperparameters and (ii) approximate predictive and sojourn densities on state and measurement space. Using simulations of three nonlinear stochastic dynamical systems, the schemes’ estimation and model identification capabilities have been demonstrated and examined in terms self-consistency. The results suggest that:

• VB-Laplace outperforms standard extended Kalman filtering, in terms of estimating of hidden-states. In particular, VB-Laplace seems to be more robust to model misspecification.
• Approximate Bayesian model comparison allows one to identify models whose structure could have generated the data. This means that the free-energy bound on log-model-evidence is not confounded by the variational approximations and remains an operationally useful proxy for model comparison.
• VB-Laplace estimators of hidden-states and model parameters seem to attain asymptotic efficiency. However, we have observed a slight but systematic underestimation of the state-noise precision hyperparameter.
• Short- and long-term prediction can be efficient, depending on the nature of the stochastic nonlinear dynamical system.

Overall, our results suggest that the VB-Laplace scheme is a fairly efficient solution to estimation, time-series prediction and model comparison problems. Nevertheless, some very specific characteristics of the proposed VB-Laplace scheme were shown to be system-specific. We discuss these properties below, along with related issues and insights.

### 5.1 On asymptotic efficiency

Asymptotic efficiency for the state-noise *per se* might be important for estimating unknown exogenous input to the system. For example, when inverting neural-mass models using neuroimaging data, retrieving the correct structure of the network might depend on explaining away external inputs. Furthermore, discovering consistent trends in estimated innovations might lead to further improvements in modelling the dynamical system. Alternative models can then be compared using the VB-Laplace approximation to the marginal likelihood as above.

We now consider an analytic interpretation of asymptotic efficiency for VB-Laplace estimators. Recall that under the Laplace approximation, the posterior covariance matrix $\Sigma_{\vartheta }$ is given by:

(58) $$\Sigma_{\vartheta }{\left(y\right)}^{-1}\approx {\langle \frac{\partial^{2}}{\partial \vartheta^{2}}\ln p\left(y,\vartheta |m\right)\rangle }_{q\left(\vartheta \right)}\text{.}$$

Therefore, its expectation under the marginal likelihood should, asymptotically, tend to the Bayesian Cramer–Rao bound:

(59) $${\left({\langle \Sigma_{\vartheta }\left(y\right)\rangle }_{p\left(y|m\right)}\right)}^{-1}\approx {\langle \Sigma_{\vartheta }{\left(y\right)}^{-1}\rangle }_{p\left(y|m\right)}\overset{\dim\left[y\right]\to \infty }{⟶}{\langle \frac{\partial^{2}}{\partial \vartheta^{2}}\ln p\left(y,\vartheta |m\right)\rangle }_{p\left(y,\vartheta |m\right)}\text{.}$$

Provided the approximate posterior density q(ϑ) converges to the true posterior density p(ϑ|y,m) with large sample sizes. For non-asymptotic regime, the normal approximation is typically more accurate for marginal distributions of components of ϑ than for the full joint distribution. Determining the marginal distribution of a component of ϑ is equivalent to averaging over all other components of ϑ; rendering it closer to normality, by the same logic that underlies the central limit theorem [51]. Therefore, the numerical evidence for asymptotic efficiency of the VB-Laplace scheme^13^ can be taken as a *post hoc* justification of the underlying variational approximations. This provides a numerical argument for extending the theoretical result of [27] on VB asymptotic convergence for conjugate-exponential (CE) models to nonlinear (non-CE) hierarchical models. Nevertheless, this does not give any prediction about the convergence rate to the likely VB-Laplace asymptotic efficiency. The Monte Carlo simulation series seem to indicate that this convergence rate might be dependent upon the system to be inverted (in our examples, the Lorenz system might be quicker than the double-well and the van der Pol systems; see Figs. 14 and 15). In other words, the minimum sample size required to confidently identify a system might strongly depend on the system itself.

In addition, VB-Laplace seems to suffer from an *underconfidence* problem: the posterior expectation of the estimation error is often over-pessimistic when compared to empirically measured estimation error. Generally speaking, free-form variational Bayesian inference on conjugate-exponential models is known to be *overconfident*[21]. This is thought to be due to the mean-field approximation, which neglects dependencies within the exact joint posterior density. However, this heuristic does not hold for non-exponential models, e.g. nonlinear hierarchical models of the sort that we are dealing with.

This underconfidence property might be due to a slight underestimation of the precision hyperparameters, which would inflate posterior uncertainty about other variables in the model. This underestimation bias of the precision hyperparameters might itself be due to the priors we have chosen (weakly informative Gamma pdf with first-order moment two orders of magnitude lower than the actual precision hyperparameters, see Tables 2 and 6). This is important, since the overall underconfidence bias (on evolution parameters) that was observed in the simulation series might be sensitive to the choice of precision hyperparameters priors.

However, this is certainly not the only effect, since this could not explain why the evolution parameter estimates of the Lorenz system are (as in the CE case) *overconfident* (see Fig. 16). Note that in this latter case, the evolution function is linear in the evolution parameters. This means that in the context of hierarchical nonlinear models, VB-Laplace might over-compensate for the tendency of variational approaches to underestimate posterior uncertainty. The subsequent underconfidence might then be due the Taylor approximation of the curvature of the log-transition density:

(60) $$\Sigma_{\theta }={\left[\frac{1}{2}\langle \alpha \rangle \underset{t}{\sum }{\frac{\partial^{2}}{\partial \theta^{2}}\langle {\left(x_{t}-f\left(x_{t-1},\theta \right)\right)}^{2}\rangle |}_{\theta =\mu_{\theta }}+\overset{-1}{\underset{\theta }{\upsilon }}\right]}^{-1}={\left[\underset{\mathrm{VB}\text{-}\mathrm{Laplace}}{\underset{︸}{\langle \alpha \rangle \underset{t}{\sum }\overset{2}{\underset{\theta =\mu_{\theta }}{\langle \frac{\partial f}{\partial \theta }\rangle |}}+\overset{-1}{\underset{\theta }{\upsilon }}}}+\underset{\mathrm{neglected}}{\underset{︸}{\langle \alpha \rangle \underset{t}{\sum }{\langle \left(x_{t}-f\left(x_{t-1},\theta \right)\right)\frac{\partial^{2}f}{\partial \theta^{2}}\rangle |}_{\theta =\mu_{\theta }}}}\right]}^{-1}\text{.}$$

Eq. (60) gives the expression for the posterior covariance matrix of the evolution parameters. When the evolution function f(x,θ) is linear in the parameters (CE case), the neglected term is zero. In this case the curvature of the log-transition density is estimated exactly, which would allow VB overconfidence to be expressed in the usual way. However, in the nonlinear case, neglecting this term will result in an overestimate of the posterior covariance. Note that underestimating α leads to an (even more) increased posterior covariance for the evolution parameters. This effect can be seen in the VB-Laplace approximation to the Lorenz sojourn distribution. This potential lack of consistency of variational Bayesian inversion of linear state-space models has already been pointed out by Wang [27]. It is possible that both effects highlighted by Eq. (60) could contribute to underconfidence in nonlinear models.

### 5.2 On time-series prediction

Our assessment of the approximate predictive and sojourn densities provided only partly satisfactory results. Overall, the VB-Laplace scheme furnishes a veridical approximation to the short-term predictive density. In addition, the long-term predictions seem to be accurate for systems that have qualitatively similar deterministic and stochastic dynamical behaviours, which is the case for both the Lorenz and the van der Pol systems, but not for the double-well system. The VB-Laplace approximation to the sojourn density relies on the ergodicity of the hidden stochastic system, which is a weak assumption for the class of systems we have considered. However, there are two classes of stochastic ergodic systems, for which the deterministic variant might also be ergodic or not. The former class of stochastic systems is called *quasideterministic*, and has a number of desirable properties [52]. The dynamical behaviour of quasideterministic systems can be approximated by small fluctuations around their deterministic trajectory (hence their name). This means that a local Gaussian approximation around the deterministic trajectory of the system will lead to a veridical approximation of the sojourn distribution. Systems are quasideterministic if and only if they are stable with respect to small changes in the initial conditions [40]. This is certainly the case for the van der Pol oscillator, which exhibits a stable limit cycle. The stochastic Lorenz system is also quasideterministic [56]. As a consequence, their VB-Laplace approximation to the stationary (sojourn) distribution is qualitatively valid. However, this is not the case for the double-well system, for which weak stochastic forces can lead to a drastic departure from deterministic dynamics [57] (e.g. phase transitions). In brief, long-term predictions based on the VB-Laplace approximations are only valid if the system is quasideterministic; i.e. if the complexity of its dynamical behaviour is not increased substantially by the stochastic effects.

### 5.3 On model comparison

In terms of model comparison, our results show that the VB-Laplace scheme could identify the structure of the hidden stochastic nonlinear dynamical system; in the sense that models that cover the dynamical structure of the hidden system are *a posteriori* the most plausible. However, the free-energy showed a slight bias in favour of more complex models: when comparing two models that could both have generated the data, the free-energy identified the model with the higher dimensionality (e.g. comparison between generic versus true Lorenz systems). This might be due to the minimum norm priors that were used for the evolution parameters. As a consequence, the structure of the true hidden system was explained by a large number of small parameters (as opposed to a small number of large parameters). Since the free-energy decreases with the Kullback–Leibler divergence between the prior and the posterior density, this “minimum norm spreading” is less costly. Importantly, this effect does not seem to confound correct model identification when models that do not cover the true structure are compared.

### 5.4 On algorithmic convergence

The variational Bayesian approach replaces the multidimensional integrals required for standard Bayesian inference by an optimization scheme. However, this optimization can also be a difficult problem, because the free-energy is a nonlinear function of the sufficient statistics of the posterior density. The VB-Laplace update rule optimizes a third-order approximation to the free-energy with respect to the sufficient statistics $\left(\mu_{i},\Sigma_{i}\right)$[28]. Note that this approximation to the free-energy comes from neglecting the contributions of fourth and higher (even) order central moments of the Gaussian approximate posterior densities. Since the latter are polynomial functions of the posterior covariance matrix $\Sigma_{i}$ (and are independent of the posterior modes $\mu_{i}$), a moment closure procedure could be used to finesse the calculation of the variational energies, guaranteeing strict convergence. However, when dealing with analytic observation and evolution functions, the series generally converge rapidly. This means that the contributions of high-order moments to the free-energy, under the Laplace approximation, become negligible. Under these conditions, marginal optimization of the variational energies almost guarantees local optimization of the free-energy.

Obviously, this does not circumvent the problem of global optimization of the free-energy. However, local convergence of the free-energy w.r.t. the sufficient statistics now reduces to local convergence of the variational energy optimization w.r.t. the modes. This is because the only sufficient statistics that need to be optimized are the first-order moments of the approximate marginal posterior densities (the second-order moments are functions of the modes; see Eq. (7)). We used a regularized Gauss–Newton scheme for the variational energy optimization, which is expected to converge under mild conditions. This convergence has been empirically observed over all our Monte Carlo simulations. However, we foresee two reasons why VB-Laplace might not converge: either the evolution or the observation functions are non-analytic or the algorithm reaches its stopping criterion too early. The first situation includes models with discrete types of nonlinearities (i.e., “on/off” switches). In this case, convergence issues could be handled by extending to switching state-space hierarchical models (see [55] for the CE case). The second situation might arise due to slow convergence rates, if the stopping criterion is based on the free-energy increment between two iterations.

### 5.5 On scalability

A key issue with Bayesian filters is scalability. It is well known that scalability is one of the main advantages of Kalman-like filters over sampling schemes (e.g. particle filters) or high-order approximations to the Kushner–Pardoux PDEs. The VB-Laplace update of the hidden-states posterior density is a regularized Gauss–Newton variant of the Kalman filter. Therefore, the VB-Laplace and Kalman schemes share the same the scalability properties.

To substantiate this claim, we analyzed the VB-Laplace scheme using basic computational complexity of matrix algebra. Assuming that arithmetic with individual elements has complexity O(1) (as with fixed-precision floating-point arithmetic), it is easy to show that the per-iteration costs (i.e. the number of computations) for the VB updates are:

(61) $$\left\{ \begin{array}{c} q\left(x\right):\underset{\mathrm{EKF}}{\underset{︸}{O\left(Tn^{3}\right)+O\left(Tpn^{2}\right)}}+\underset{\mathrm{mean}\text{-}\mathrm{field terms}}{\underset{︸}{O\left(Tn_{\theta }n^{3}\right)+O\left(Tn^{2}\overset{2}{\underset{\theta }{n}}\right)+O\left(Tnpn_{\varphi }^{2}\right)+O\left(Tn_{\varphi }pn^{2}\right)}}\\ q\left(\alpha \right):O\left(Tn^{2}\right)+O\left(T\overset{3}{\underset{\theta }{n}}\right)+O\left(Tn_{\theta }n^{3}\right)+O\left(Tn^{2}\overset{2}{\underset{\theta }{n}}\right)\\ q\left(\sigma \right):O\left(Tp^{2}\right)+O\left(Tp\overset{2}{\underset{\varphi }{n}}\right)+O\left(T\overset{3}{\underset{\varphi }{n}}\right)+O\left(Tn_{\varphi }pn^{2}\right)+O\left(Tnp\overset{2}{\underset{\varphi }{n}}\right)\\ q\left(\theta \right):O\left(T\overset{3}{\underset{\theta }{n}}\right)+O\left(Tn_{\theta }n^{3}\right)+O\left(Tn^{2}\overset{2}{\underset{\theta }{n}}\right)\\ q\left(\varphi \right):O\left(T\overset{3}{\underset{\varphi }{n}}\right)+O\left(Tp\overset{2}{\underset{\varphi }{n}}\right)+O\left(Tn_{\varphi }pn^{2}\right)+O\left(Tnp\overset{2}{\underset{\varphi }{n}}\right)\text{.} \end{array}\right.$$

This derives from the sparsity of the mean-field terms, which rely on Kronecker products with identity matrices (see Eqs. (29), (31) and (34)). It can be seen that the per-iteration cost is the same as a Kalman filter; i.e., it grows as $O\left(n^{3}\right)$, where n is the number of hidden-states.

In terms of memory, the implementation of our VB scheme has the following matrix storage requirements: $nT\left(6+5n\right)+n_{\theta }\left(1+n_{\theta }\right)+n_{\varphi }\left(1+n_{\varphi }\right)$, which is required for the calculation of the posterior covariance matrices (see Eqs. (29), (31) and (34)). This computational load is similar to a Kalman filter; i.e., it grows as $O\left(n^{2}\right)$. Overall, this means that the VB-Laplace scheme inherits the scalability properties of the Kalman filter.

### 5.6 On influence of noise

In the Monte Carlo simulation series we presented, we did not assess the response of the VB-Laplace scheme to a systematic variation of noise precision. This was justified by our main target application, i.e. neuroimaging data (EEG/MEG and fMRI) analysis, for which the SNR is known (see e.g., [53]).

In addition, we have also fixed the state-noise precision hyperparameter. This is because a subtle balance between drift and state-noise is required for stochastic dynamical systems to exhibit “interesting” properties, which would disappear in both low- and high-noise situations. For example, the expected time interval between two transitions of the double-well system is proportional to the state-noise precision (see e.g. [54]). As a consequence, the low-noise double-well system will hardly show any transition. In contradistinction, the high-noise double-well system looks like white noise, because the drift term has no significant influence on the dynamics anymore. Therefore, local and global oscillations co-occur only within a given range of state-noise precision (stochastic resonance).

Nevertheless, a comprehensive assessment of the behaviour of the VB-Laplace scheme would require varying the precision of both the measurement and the state-noise precisions. Preliminary results (not shown) seem to indicate that the VB-Laplace scheme does not systematically suffer from over- or under-fitting, even in the weakly informative precision prior case. However, no formal conclusions can yet be drawn onto the influence of high noise on the VB-Laplace scheme, which could potentially be a limiting factor for particular applications.

## 6 Conclusion

In this paper, we have presented an approximate variational Bayesian inference scheme to estimate the hidden-states, parameters, and hyperparameters of dynamic nonlinear causal models. We have also assessed its asymptotic efficiency, prediction ability and model selection performances using decision theoretic measures and extensive Monte Carlo simulations. Our results suggest that variational Bayesian techniques are a promising avenue for solving complex inference problems that arise from structured uncertainty in dynamical systems.

## Figures and Tables

**Table 1:** ODEs of three chaotic dynamical systems.

Double-well	$\overset{˙}{x}=\left(\begin{array}{c} x_{2}\\ -2\left(x_{1}-\theta_{1}\right){\left(x_{1}-\theta_{2}\right)}^{2}-2{\left(x_{1}-\theta_{1}\right)}^{2}\left(x_{1}-\theta_{2}\right)-\theta_{3}x_{2} \end{array}\right)$
Lorenz	$\overset{˙}{x}=\left(\begin{array}{c} \theta_{2}\left(x_{2}-x_{1}\right)\\ x_{1}\left(\theta_{1}-x_{3}\right)-x_{2}\\ x_{1}x_{2}-\theta_{3}x_{3} \end{array}\right)$
van der Pol	$\overset{˙}{x}=\left(\begin{array}{c} x_{2}\\ \theta_{1}\left(1-x_{1}^{2}\right)x_{2}-x_{1} \end{array}\right)$

**Table 2:** Parameters of the generative model for the three simulated dynamical systems.

		Double-well	Lorenz	van der Pol
Measurement-noise precision	Simulated	$\sigma =10^{2}$	$\sigma =10^{2}$	$\sigma =10^{1}$
	Prior pdf	$\varsigma_{\sigma }=10^{2},\upsilon_{\sigma }=1$	$\varsigma_{\sigma }=10^{5},\upsilon_{\sigma }=10^{3}$	$\varsigma_{\sigma }=10^{2},\upsilon_{\sigma }=1$
System-noise precision	Simulated	$\alpha =10^{2}$	$\alpha =10^{2}$	$\alpha =10^{2}$
	Prior pdf	$\varsigma_{\alpha }=1,\upsilon_{\alpha }=1$	$\varsigma_{\alpha }=10^{-2},\upsilon_{\alpha }=10^{-2}$	$\varsigma_{\alpha }=10^{-2},\upsilon_{\alpha }=10^{-2}$
Evolution parameters	Simulated	$\theta ={\left(3,-2,3/2\right)}^{T}$	$\theta ={\left(28,10,8/3\right)}^{T}$	θ=1
	Prior pdf	$\varsigma_{\theta }=0_{3},\upsilon_{\theta }=10^{2}I_{3}$	$\varsigma_{\theta }=0_{3},\upsilon_{\theta }=10I_{3}$	$\varsigma_{\theta }=0,\upsilon_{\theta }=10^{2}$
Initial conditions	Simulated	$∼N\left({\left[5,0\right]}^{T},10^{-3}I_{2}\right)$	$∼N\left(1_{3},10^{-1}I_{3}\right)$	$∼N\left(0_{2},I_{2}\right)$
	Prior pdf	$\varsigma_{0}={\left[5,0\right]}^{T},\upsilon_{0}=10^{-3}I_{2}$	$\varsigma_{0}=1_{3},\upsilon_{0}=10^{-1}I_{3}$	$\varsigma_{0}=0_{2},\upsilon_{0}=I_{2}$
Observation function	b	0.5	0.2	5
	$G_{0}$	50	50	50

**Table 3:** Monte Carlo average log-SEL for the VB-Laplace, EKF1 and EKF2 approaches for three different stochastic systems.

	Double-Well	Lorenz	van der Pol
VB-Laplace	3.32	4.24	4.02
EKF1	8.80 a	8.58 a	13.9 a
EKF2	3.35	4.19 a	4.39 a

a Indicates a significant difference relative to the corresponding VB-Laplace SEL score (one-sample paired t-test, 5% confidence level, df=49). The grey cells of the table indicate which of the three approaches (VB-Laplace, EKF1 or EKF2) were best, in terms of efficiency.

**Table 4:** Prior density over the evolution parameters for the “generic” model for the three dynamical systems.

	Double-well	Lorenz	van der Pol
Evolution parameters prior pdf	$\varsigma_{\theta }=0_{10},\upsilon_{\theta }=I_{10}$	$\varsigma_{\theta }=0_{27},\upsilon_{\theta }=10I_{27}$	$\varsigma_{\theta }=0_{10},\upsilon_{\theta }=10I_{10}$

**Table 5:** Monte Carlo averages of model accuracy indices: free-energy, goodness-of-fit (SSE) and estimation loss (SEL) as functions of the class stochastic systems.

		Double-well	Lorenz	van der Pol
Free-energy	Native model	−1.98×10^3^^a^	1.04×10^6^	−5.55× 10^2^^a^
	Generic model	−3.04×10^3^	1.05×10^6^^a^	−8.83× 10^2^
log SSE	Native model	0.53 a	0.37 a	3.58
	Generic model	0.60	0.72	2.93 a
log-SEL	Native model	3.32 a	4.24 a	4.00 a
	Generic model	6.29	6.98	5.01

a Indicates a significant difference between the true and generic models (one-sample paired t-test, 5% confidence level, df=49). Grey cells indicate which of the two models (true or generic) are best with respect to the three indices.

**Table 6:** Parameters of the generative model for the three dynamical systems.

		Double-well	Lorenz	van der Pol
Measurement-noise precision	Simulated	$\sigma =10^{2}$	$\sigma =10^{2}$	$\sigma =10^{2}$
	Prior pdf	$\varsigma_{\sigma }=10^{2},\upsilon_{\sigma }=1$	$\varsigma_{\sigma }=10^{2},\upsilon_{\sigma }=1$	$\varsigma_{\sigma }=10^{2},\upsilon_{\sigma }=1$
System-noise precision	Simulated	$\alpha =10^{3}$	$\alpha =10^{2}$	$\alpha =10^{3}$
	Prior pdf	$\varsigma_{\alpha }=1,\upsilon_{\alpha }=1$	$\varsigma_{\alpha }=10^{-2},\upsilon_{\alpha }=10^{-2}$	$\varsigma_{\alpha }=10^{-2},\upsilon_{\alpha }=10^{-2}$
Evolution parameters	Simulated	$\theta ={\left(3,-2,3/2\right)}^{T}$	$\theta ={\left(28,10,8/3\right)}^{T}$	θ=1
	Prior pdf	$\varsigma_{\theta }=0_{3},\upsilon_{\theta }=10^{2}I_{3}$	$\varsigma_{\theta }=0_{3},\upsilon_{\theta }=10I_{3}$	$\varsigma_{\theta }=0,\upsilon_{\theta }=10$
Initial conditions	Simulated	$∼N\left({\left[5,0\right]}^{T},10^{-3}I_{2}\right)$	$∼N\left(1_{3},I_{3}\right)$	$∼N\left(0_{3},10^{2}I_{2}\right)$
	Prior pdf	$\varsigma_{0}={\left[5,0\right]}^{T},\upsilon_{0}=10^{-3}I_{2}$	$\varsigma_{0}=1_{3},\upsilon_{0}=I_{3}$	$\varsigma_{0}=0_{2},\upsilon_{0}=I_{2}$

## Footnotes

1 Note that *filtering* techniques provide the instantaneous posterior density, (i.e. the posterior density given observed time-series data so far) as opposed to *smoothing* schemes, which cannot operate on-line, but furnish the full posterior density (given the complete time-series data).

2 In this article, we refer to parameters governing the second-order moments of the probability density functions as (variance or, reciprocally, precision) *hyperparameters*.

3 Note that all these quantities are the negative of their thermodynamic homologues.

4 The class of decision theoretic problems (i.e. hypothesis testing) is treated as a model comparison problem in a Bayesian framework.

5 One can apply any arbitrary nonlinear transform to the parameters to implement an implicit probability integral transform.

6 The Markov blanket of a node in a directed acyclic graph (of the sort given in Fig. 1) comprises the node’s parents, children and parents of those children.

7 For both hidden-states and initial conditions, we halve the size of the Gauss–Newton update until their respective variational energy increases.

8 To compare different variables and systems, we used a relative squared error loss (*RSEL*), defined as:

$$RSEL\left(\vartheta \right)=\underset{i}{\sum }{\left(\vartheta_{i}-{\overset{ˆ}{\vartheta }}_{i}\right)}^{2}/\overset{2}{\underset{i}{\vartheta }}\text{.}$$

We report this measure in log space as a function of T i.e., ln(RSEL(T)), such that ln(RSEL(T))≤−2 means that the relative estimation error is smaller than 10^−1^ ($0.9\;\vartheta_{i}\leq {\overset{ˆ}{\vartheta }}_{i}\leq 1.1\;\vartheta_{i}$).

9 Note that the relationship between RSEL and T depicted in Fig. 14 might not, strictly speaking, appear monotonic (cf., e.g., the Lorenz evolution parameters). This is likely to be due to finite size effects in the Monte Carlo simulation series (50 samples per value of T). However, the rate at which the VB-Laplace reaches the asymptotic regime might be different for the systems considered (see Section 5 “on asymptotic efficiency”).

10 Strictly speaking, $q\left(x_{0}\right)$ also depends on q(α,θ).

11 A cyclostationary system is such that the sufficient statistics of its predictive density are periodic. It can be thought of as an ergodic process that constitutes multiple interleaved stationary processes [50].

12 Note that the bimodality of the predictive density does not imply bimodality of the posterior density.

13 The Monte Carlo simulations provide us with a sampling approximation to the left-hand term of Eq. (55) (sampling averages of the squared error loss, see Figs. 8 and 9) given model m.
